# Thymosin β4 Is an Endogenous Iron Chelator and Molecular Switcher of Ferroptosis

**DOI:** 10.3390/ijms23010551

**Published:** 2022-01-04

**Authors:** Joanna I. Lachowicz, Giusi Pichiri, Marco Piludu, Sara Fais, Germano Orrù, Terenzio Congiu, Monica Piras, Gavino Faa, Daniela Fanni, Gabriele Dalla Torre, Xabier Lopez, Kousik Chandra, Kacper Szczepski, Lukasz Jaremko, Mitra Ghosh, Abdul-Hamid Emwas, Massimo Castagnola, Mariusz Jaremko, Ewald Hannappel, Pierpaolo Coni

**Affiliations:** 1 Department of Medical Sciences and Public Health, University of Cagliari, Cittadella Universitaria, 09042 Monserrato, Italy; lachowicz@unica.it (J.I.L.); terenzio.congiu@unica.it (T.C.); monipiras@hotmail.com (M.P.); gavinofaa@gmail.com (G.F.); daniela.fanni@unica.it (D.F.); ppconi@tiscali.it (P.C.); 2 Department of Biomedical Sciences, University of Cagliari, Cittadella Universitaria, 09042 Monserrato, Italy; 3 Department of Surgical Science, OBL Oral Biotechnology Laboratory, University of Cagliari, 09124 Cagliari, Italy; sarafais79@gmail.com (S.F.); gerorru@gmail.com (G.O.); 4 Kimika Fakultatea, Euskal Herriko Unibertsitatea UPV/EHU, Donostia International Physics Center (DIPC), P.K. 1072 Donostia Euskadi, 20080 San Sebastian, Spain; gdtorre1@gmail.com (G.D.T.); xabier.lopez@ehu.es (X.L.); 5 Smart-Health Initiative (SHI) and Red Sea Research Center (RSRC), Division of Biological and Environmental Sciences and Engineering (BESE), King Abdullah University of Science and Technology (KAUST), Thuwal 23955-6900, Saudi Arabia; kousik.chandra@kaust.edu.sa (K.C.); kacper.szczepski@kaust.edu.sa (K.S.); lukasz.jaremko@kaust.edu.sa (L.J.); mitra.ghosh@kaust.edu.sa (M.G.); 6 Core Labs, King Abdullah University of Science and Technology (KAUST), Thuwal 23955-6900, Saudi Arabia; abdulhamid.emwas@gmail.com; 7 Institute of Chemistry of Molecular Recognition, National Research Council (Consiglio Nazionale delle Ricerche), 00185 Rome, Italy; maxcastagnola@outlook.it; 8 Laboratory of Proteomics and Metabolomics, IRCCS, Santa Lucia Foundation, 00143 Rome, Italy; 9 Institute of Biochemistry, Friedrich-Alexander-University Erlangen-Nuremberg, 91058 Erlangen, Germany; ewald.hannappel@fau.de

**Keywords:** thymosine beta 4, ferroptosis, metal chelation, TEM, mRNA, molecular dynamics, NMR

## Abstract

Thymosin β4 (Tβ4) was extracted forty years agofrom calf thymus. Since then, it has been identified as a G-actin binding protein involved in blood clotting, tissue regeneration, angiogenesis, and anti-inflammatory processes. Tβ4 has also been implicated in tumor metastasis and neurodegeneration. However, the precise roles and mechanism(s) of action of Tβ4 in these processes remain largely unknown, with the binding of the G-actin protein being insufficient to explain these multi-actions. Here we identify for the first time the important role of Tβ4 mechanism in ferroptosis, an iron-dependent form of cell death, which leads to neurodegeneration and somehow protects cancer cells against cell death. Specifically, we demonstrate four iron^2+^ and iron^3+^ binding regions along the peptide and show that the presence of Tβ4 in cell growing medium inhibits erastin and glutamate-induced ferroptosis in the macrophage cell line. Moreover, Tβ4 increases the expression of oxidative stress-related genes, namely BAX, hem oxygenase-1, heat shock protein 70 and thioredoxin reductase 1, which are downregulated during ferroptosis. We state the hypothesis that Tβ4 is an endogenous iron chelator and take part in iron homeostasis in the ferroptosis process. We discuss the literature data of parallel involvement of Tβ4 and ferroptosis in different human pathologies, mainly cancer and neurodegeneration. Our findings confronted with literature data show that controlled Tβ4 release could command on/off switching of ferroptosis and may provide novel therapeutic opportunities in cancer and tissue degeneration pathologies.

## 1. Introduction

Ferroptosis is an iron-dependent oxidative form of cell death that is morphologically, biochemically, and genetically distinct from other non-apoptotic cell death processes [1]. The latest definition of ferroptosis was presented in 2018 by the Nomenclature Committee on Cell Death (NCCD) [2] as “a form of regulated cell death (RCD) initiated by oxidative perturbations of the intracellular microenvironment that is under constitutive control by GPX4 and can be inhibited by iron chelators and lipophilic antioxidants. Ferroptosis initiates with lipid peroxidation due to ROS generation and iron availability”. Ferroptosis leads to morphological changes with a necrotic morphotype (e.g., predominant mitochondrial shrinking, an electron-dense ultrastructure, reduced/disappeared cristae, and ruptured outer mitochondrial membrane) [2]. Ferroptotic cell death is thought to require mutation of the oncogenes HRAS, KRAS, or BRAF, and is caused by lethal iron-dependent lipid peroxidation [3,4]. To provoke cell death, ferroptosis likely requires continuous iron-dependent ROS production over a prolonged period, which can be observed in neuronal, cardiac, and hepatic cells when exposed to high iron concentrations in neurodegeneration pathology [5], ischemic conditions [6], and hepatic chromatosis [7], respectively.

Iron, a highly redox-reactive element, is required in a variety of biological processes. Cancer cells develop different mechanisms both to uptake high quantities of iron and to protect themselves against iron toxicity [8]. Activation of the ferroptosis suppresses tumor cell growth and successively leads to cell death. The controlled on/off switching of ferroptosis may provide novel therapeutic opportunities in pathologies of cancer and tissue degeneration, respectively.

**Scheme 1:**
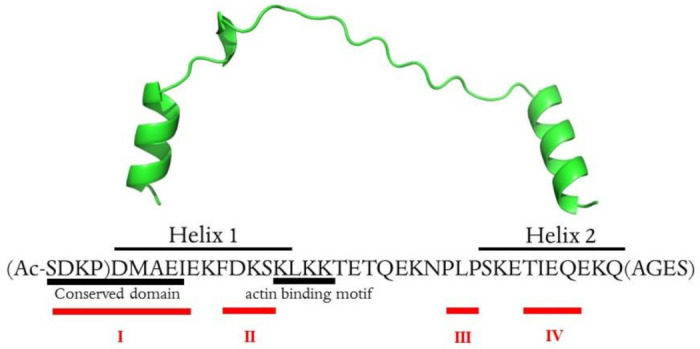
Primary sequence and 3D structure of Tβ4 (PDB: 4PL7). Amino acids in parentheses were not defined by the X-ray structure and were added using Modeller v. 10.0 following the proper secondary structure elements (alpha helix). The metal-binding sites (I–IV) are signed in red. The scheme was created by Joanna Izabela Lachowicz, University of Cagliari. This image is original and designed specifically for the targeted publication. It should not be cropped, distorted, or in any way edited without the expressed consent of Research Publication Services.

Thymosin β4 (Tβ4; Scheme 1) is a G-actin binding protein with different functions in the human body, including blood clotting [9], tissue regeneration [10], angiogenesis [11], prenatal and early childhood development [12], and tumor metastasis [13]. Tβ4 is also involved in anti-inflammatory and neurodegenerative processes [14]. At the subcellular level, Tβ4 is localized in the cytoplasm [15] and/or nucleus [16] and can be translocated between compartments after induction [16,17]. Thymosin β4-overexpression is correlated with different human cancer types, e.g., pancreatic [18] and colorectal cancer [19], and Tβ4 is considered a potential molecular target for anticancer therapy. Indeed, Tβ4 gene silencing decreases stemness and invasiveness in glioblastoma [20] and suppresses proliferation and invasion of non-small cell lung cancer cells [21].

The exact mechanisms of Tβ4 action are still unknown. Recently, we hypothesized that the regulatory function of Tβ4 in numerous physiological processes is correlated with its potential essential metal-binding ability [22]. Here, we discuss for the first time the formation of a metal complex with Tβ4 and its biological importance in ferroptosis. We observed that an increased concentration of Tβ4 can effectively inhibit the ferroptosis process, a mechanism that is used by cancer cells with enhanced Tβ4 expression. The inhibition of ferroptosis by augmenting extracellular concentrations of Tβ4 can stop cell degeneration, and such a strategy could be used in therapy.

## 2. Results

### 2.1. Thymosin β4 Metal Coordination

Thymosin β4 is a 43 amino acid peptide with an acetylated N-terminus (Scheme 1). It is an acidic peptide (pI 5.1) due to the presence of 11 amino acids with carboxylic groups in the side chain, which are the potential metal binding sites [23]. Moreover, nine lysine residues in the primary sequence have a high affinity to transition metal ions [23]. Thymosin β4 has no secondary structure in pure water, while in aqueous solution with the addition of TFE (or HFP), two alpha-helixes are formed in the 5–17 and 30–39 regions [24].

To our knowledge, no experimental or molecular dynamics study of the formation of essential metal complexes has been performed with Tβ4, despite there being numerous potential metal-binding sites. In order to investigate the metal-binding sites in the Tβ4 peptide, we chose nuclear magnetic resonance (NMR) techniques, which deliver valuable structural data of metal complexes [25].

The interaction of iron ions with Tβ4 in water was investigated in a residue-specific manner by monitoring of 2D ^13^C-^1^H HSQC (heteronuclear single quantum coherence) NMR (Figures S1–S3, S5 and S6), focused on the aliphatic region. The overlay of the HSQC data (Figures S2, S3, S5 and S6) showed that metal/peptide interactions are specific while formed in specific regions of the peptide, but there is no structural change upon interaction with iron^2+^ nor iron^3+^ ions.

The presence of metal ions in the peptide solution leads to the lowering of the peptide signals. The comparative analysis of the peptide signal pattern with and without metal ions permits the isolation of amino acids affected by the interactions with the metal ions (Figures S4 and S7). In the thymosin solution containing iron^2+^ and iron^3+^ ions, eighteen and fifteen residues, respectively, showed a major drop in intensity (Table 1). Since iron^3+^ is paramagnetic, we observed greater overall signal broadening of the residues, with the aforementioned residues showing major drops in intensity.

Circular dichroism (CD) structural analysis of free Tβ4 in water showed that the secondary structure is mainly unorganized, and only 20% could be associated with the helical form. The addition of iron ions to the peptide solution did not significantly change the secondary structure (Figure S9).

In general, NMR data precision is limited by the presence of paramagnetic species and does not permit detailed coordination analysis with iron ions. For this purpose, Al^3+^ ions, which form similar stoichiometry and geometry complexes as Fe^3+^, were used to model Fe^3+^/Tβ4 complexes.

**Figure 1:**
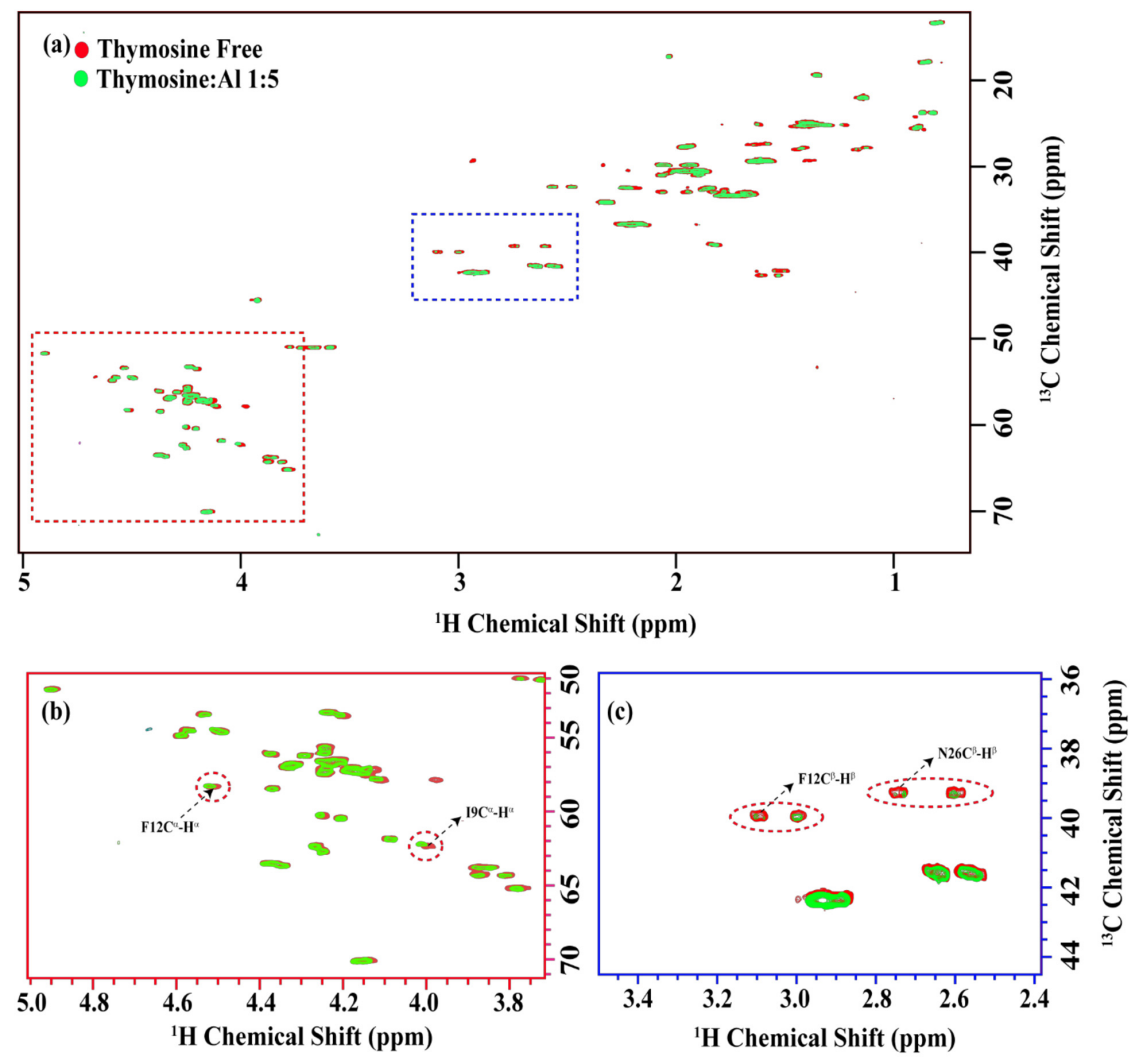
(**a**) The overlay of the two-dimensional ^13^C-^1^H HSQC spectra in free form (red) and with thymosin: Al^3+^ ratio 1:5 (green). The alpha and beta proton regions are highlighted in the red and blue dotted rectangles in (**a**) and are presented in (**b**,**c**), respectively. Noticeable chemical shift changes were observed for F12, I9 as indicated by red circles in (**b**). Otherwise, the chemical shifts remain the same, with only some decrease in the intensities, as highlighted by the red circles in (**c**).

The analysis of the overlay HSQC data (Figure 1) showed that metal/peptide interactions are specific while formed in specific regions of the peptide, but there were no significant changes in the secondary structure of thymosin upon interaction with Al**^3+^** ions.

**Figure 2:**
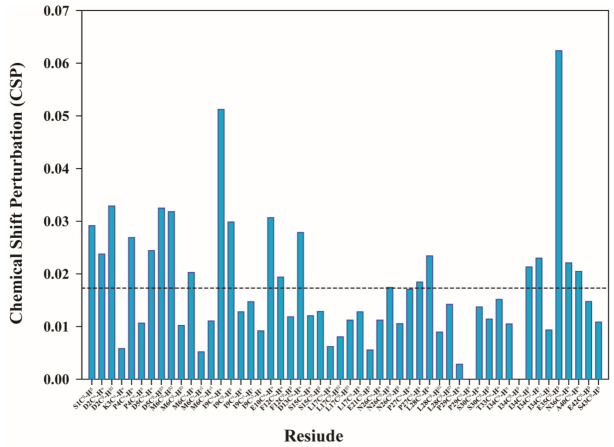
The chemical shift perturbation (CSP) plot for the 48 distinct peaks from the overlay of the two-dimensional ^13^C-^1^H HSQC spectra in free form and with thymosin:Al^3+^ ratio 1:5.The formula used for CSP is Sqrt [1/2(δ_H_^2^ + α ∗ δ_13C_^2^)] as suggested by Willamson [26]. The α was kept 0.3.

The chemical shift perturbation (CSP) was calculated considering the free peptide form and 1:5 (peptide:metal) as the maximum stoichiometry leading to the reduction of the peptide’s peak intensities and no further chemical shift changes. Only 48 isolated peaks were considered for calculating the CSP and intensity analysis. Analysis of the CSP plot (Figure 2) showed that Al^3+^ ions interact with N-terminal residues of Tβ4. The residues that most likely interact with the metal ion are listed in Table 1.

Figure 2 shows that residues Ile^9^ and Gln^35^ presented noticeable changes upon interaction with Al^3+^, however, the CSP value is too small to define the metal/peptide interaction as strong and with high affinity. Moreover, the changes in Ile^9^ and Gln^35^ signals could be associated with peptide bending upon metal coordination by adjacent residues (with carboxylic groups in amino acids side chains), rather than participation in the metal coordination core. The plot of signal intensities as a function of the Tβ4:metal ratio showed a consistent decrease for most of the residues in 1D (Figure S12) and in 2D (Figure S13). However, it is important to note that the intensity of 1D spectra is directly proportional only to the population, while the 2D spectra intensity is modulated by many different factors.

The diffusion NMR data at the different metallopeptide ratios showed the presence of only one species with a diffusion constant 1.25 × 10 ^10^ m^2^/s, thus, the formation of the peptide aggregates upon metal binding can be excluded.

The NMR data analysis showed that metal ions interact with numerous amino acid residues, and four metal-binding sites could be distinguished within the Tβ4 structure (namely I–IV, Scheme 1, Table 1). The binding modes within four metal binding sites were modeled with the density functional theory (DFT) calculation.

Tβ4 contains 11 acidic residues: eight glutamate and three aspartate residues [27]. According to Pearson’s hard and soft acids and bases (HSAB) principle, hard metal ions (Fe^3+^ and Al^3+^) show a preference for binding hard bases, such as the negatively charged carboxylate side chains of glutamate and aspartate amino acids. Although there can be other donors to hard metal ions, such as backbone carbonyl and amide groups of proteins, by virtue of the partial covalent nature of the coordination bond, these donors are less competitive. Indeed, we recently demonstrated, from a thermodynamic perspective, that the preferred binding sites of Al^3+^ in proteins are the negatively charged side chains of amino acids rather than the backbone carbonyl and amide groups [28].

The chemical shift perturbation of the NMR data showed that the main changes in chemical shifts upon Al^3+^ addition arise in two different areas of the protein: within the N-terminal segment (metal-binding sites I and II) and the C-terminal segment (metal-binding sites III and IV).

The conformational analyses were prepared in order to build 3D models of the Al^3+^-Tβ4 complex and obtain some reliable initial configurations of this hard metal ion bound to Tβ4. According to the literature, Tβ4 in water solution exists mainly in a disordered state and it is unclear whether secondary structure elements such as extended helices in the N- and C-terminus of the protein arise upon interaction with other partners such as actin [22]. Thus, in the starting structure, the Tβ4 sequence was considered “unfolded”, without main secondary structure elements (helices and sheets).

Three major binding modes for the Al^3+^-Tβ4 system were obtained (Table 2): first, the metal interacting with both the N- and C-terminus of the protein (Tβ4^N-C^); second with the metal-bound to the N-terminal and in the middle of the protein (Tβ4^N-mid^); third with the metal-bound only in the N-terminus of the protein (whereas the rest of the sequence remains highly disordered; Tβ4^N-N^). Interestingly, all the three binding modes featured four negatively charged residues (side chains of either ASP or GLU) with a net charge of −4 surrounding the metal center, a situation that is coherent with the one proposed by Dudev and Lim [29], who stated that the optimal number of acetate-type donors in a metal-binding site is equal to q + 1, where q is the charge of the metal when first coordination shell residues are taken into account.

**Figure 3:**
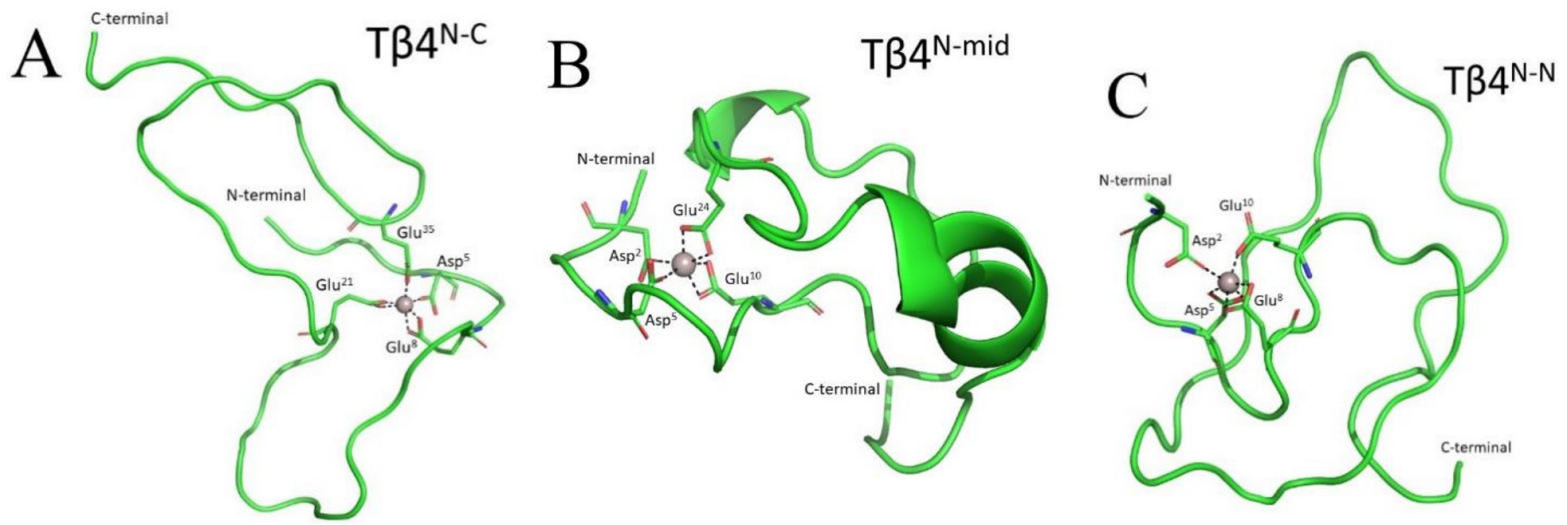
Representative structures of (**A**) Tβ4^N-C^; (**B**) Tβ4^N-mid^ and (**C**) Tβ4^N-N^. Figure was created by Gabriele dalla Torre, Euskal Herriko Unibertsitatea UPV/EHU. This image is original and designed specifically for the targeted publication. It should not be cropped, distorted, or in any way edited without the expressed consent of Research Publication Services.

In Tβ4^N-C^ binding mode, the metal is bound to GLU^8^ and GLU^21^ in a bidentate fashion, and to ASP^5^ and GLU^35^ by a single oxygen donor (Figure 3A). The six oxygen donors coordinate Al^3+^ through an octahedral geometry, with O-Al-O angles ≥150 degrees. During the simulation, after 3 μs, the carboxylate side chain of GLU^35^ coordinates the metal with both oxygen donors, whereas the side chain of GLU^21^ switches to a monodentate binding mode. The original state is then restored after 4 μs up to the end of the simulation. Overall, the TB4^N-N^ protein-metal complex remains stable for the entire course of the simulation, and no other residues nor water molecules enter the first coordination shell of the metal.

The Tβ4^N-mid^ binding mode is characterized by Al^3+^ ions coordinated to the side chains of ASP^2^ and ASP^5^ in a monodentate fashion and to the side chains of GLU^10^ and GLU^24^ in a bidentate fashion (Figure 3B). Moreover, in this case, there is no change in the coordination pattern of Tβ4^N-mid^ during the entire simulation time of 5 μs.

Finally, the Tβ4^N-N^ binding mode displayed an octahedral geometry, in which the first coordination shell of the metal is filled by one oxygen donor from the side chains of ASP^5^ and GLU^8^ and two oxygen donors from the side chains of ASP^2^ and GLU^10^ (Figure 3C). The metal center maintains this binding mode for the whole simulation.

Structural characterization revealed that Tβ4^N-N^ displays the highest degree of disorder less compaction rate with the average root mean square deviation (RMSD) and radius of gyration (Rg) of 1.07 and 1.29, respectively, compared to Tβ4^N-mid^ (0.89 and 1.11, respectively) and Tβ4^N-C^ (0.92 and 1.15, respectively) as reported in Table S1. This is coherent with the fact that in the case of Tβ4^N-mid^ and Tβ4^N-C^, the N- and C- termini are closer due to their respective metal binding modes that involve residues either in the medial or distal region of the protein, as also highlighted by their shorter end to end distance compared to TB4^N-C^ (Table S1). The average amount of intramolecular hydrogen bonds are similar for Tβ4^N-C^ and Tβ4^N-N^ and slightly higher for Tβ4^N-mid^ (Table S1); most populated H-bonds and salt bridges for each binding mode are reported in Table 2.

As can be deduced from Table S2, all three metal-Tβ4 complexes exist mainly as random coil structures; the prevalent structural motifs are bends and turns, consistent with the disordered nature of Tβ4. Interestingly, Tβ4^N-C^ and Tβ4^N-mid^ display a similar β-sheet content (16.8 and 17.6%, respectively), whereas Tβ4^N-N^ shows a much lower content of this structural motif (2.5%). On the other hand, the latter binding mode displays a higher helicity propensity compared with the other two, in particular, it features 94.1% of the 3_10_-helix motif. Such a situation could be due to the fact that the metal-binding activity of Tβ4^N-N^ is confined to the N-terminal segment of the protein, while the remaining sequence is highly disordered and may increase the propensity to form helical patterns in its C-terminal regions.

All three binding modes are quite stable across the 5 μs time scale. We also observed that Tβ4 binding to aluminum could affect the overall secondary structure pattern of the thymosin. Since both glutamate and aspartate residues are the preferred acidic groups of hard metals, the three binding modes characterized herein could fit either Al^3+^ or Fe^3+^ metal ions.

### 2.2. Thymosin β4 and Ferroptosis

Macrophages, together with hepatocytes, play key roles in iron metabolism by mediating iron storage and recycling [30]. In hereditary hemochromatosis (HH), a representative disease that causes iron overload, hepatocytes are the first site of iron accumulation [31,32]. Recent studies [33,34] have shown that pathological iron overload in mouse macrophage cell line J774 induces ferroptosis and subsequent cell destruction. Therefore, we used J774 cells as a model to study the importance of macrophages in iron metabolism [35] and their consequent susceptibility to ferroptosis.

Ferroptosis was primarily characterized by condensed mitochondrial membrane densities and smaller mitochondria, as well as diminished or absent mitochondria crista and ruptured outer membrane [36]. Recent studies divided mitochondrial morphological alterations into three categories according to whether the mitochondria were fragmented or not, and whether they accumulated around the nucleus. Cells with a network of elongated mitochondria were assigned to category I, uniform-distributed fragmented mitochondria in cells were classified as category II, and cells with fragmented mitochondria mainly accumulating around the nucleus were classified as category III [36,37,38,39]. Mitochondrial morphological changes and metabolic regulation, especially the energetic, iron and aliphatic acid metabolism involved in ferroptosis, were extensively described in a recent review [40].

**Figure 4:**
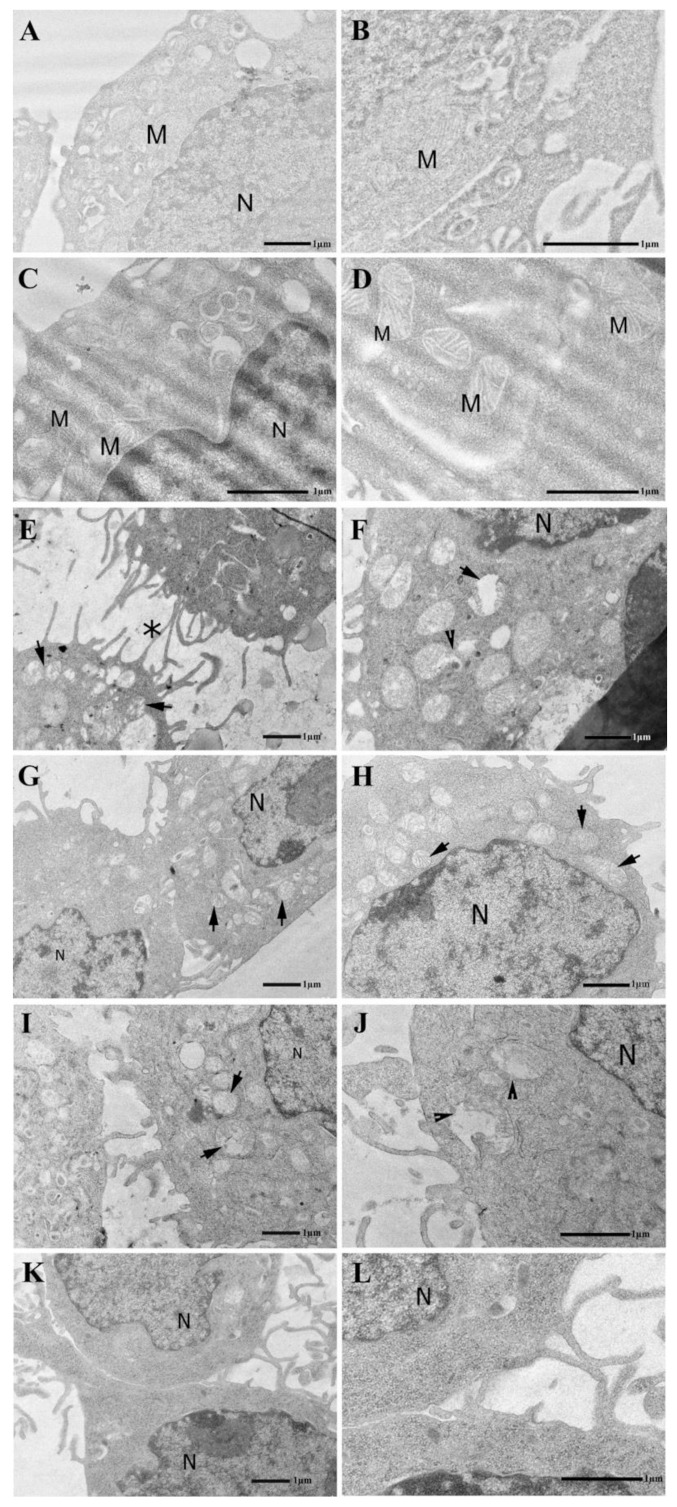
Electron micrographs of J774cells. (**A**,**B**) The Control group displays a well-preserved ultrastructure. (**C**,**D**) Administration of Tβ4 (10 μM, 24 h) does not appear to alter cell morphology. Note in both control and Tβ4 treated samples the presence of several mitochondria showing a continuous mitochondria membrane and well-organized cristae. M = mitochondria, N = nucleus. (**E**,**F**) Glutamate treatment (5.5 mM, 24 h) induces numerous cellular alterations that affect especially the mitochondria (arrows). Initially, it appears that the membranes of the mitochondria open up, letting the contents pour out (arrowhead). Note the presence of tunneling nanotubes connecting adjacent cells (asterisk). (**G**,**H**) After Tβ4 administration (10 μM, 20 h), the cytoplasmic compartment displays more preserved cell structures. Mitochondria show a continuous mitochondrial membrane and partially reorganized cristae. N = nucleus. (**I**,**J**) Erastin treatment (0.5 μM; 24 h) induces numerous cellular alterations that affect especially the mitochondria (arrows). Mitochondria are frequently observed with ruptured mitochondrial membrane and altered cristae (arrowheads). (**K**,**L**) After Tβ4 administration (10 μM, 20 h), the cytoplasmic compartment displays more preserved cell structures associated with an evident decrease in lysosomal activity. N = nucleus.

For a more detailed characterization of glutamate- and erastin-induced ferroptosis in J774 cells, including Tβ4 inhibition, we evaluated the morphological changes by TEM microscopy. The results obtained from this ultrastructural analysis are summarized in Figure 4 and Figure S19, showing cells from different experimental groups. In the control group (Figure 4A,B), the J774 cells appeared normal at the ultrastructural level. They were mainly characterized by a well-developed Golgi apparatus, rough endoplasmic reticulum, and the presence of numerous mitochondria. The nuclear compartment was separated from the cytoplasm by the double membrane of the nuclear envelope, and the cells appeared to be in interphase due to the presence of both heterochromatin and euchromatin. The presence of densely packed chromatin and the absence of the nuclear envelope represented the main features of mitotic cells, which were often observed in control samples (Figure S19).

Administration of Tβ4 (Figure 4C,D) did not appear to alter cell morphology: samples displayed structural integrity of all their cellular compartments and organelles, including mitochondria.

Following glutamate (Figure 4E,F) and erastin (Figure 4I,J) treatments, the cellular morphology of the J774 cells was dramatically altered. Specific ultrastructural alterations were mainly observed in the cytoplasmic compartment, where numerous mitochondria were found accumulating around the nucleus. These cellular organelles appeared drastically altered, showing mitochondrial membrane rupture and evident cristae disorganization. Moreover, the presence of lysosomal activity and disruption of the Golgi apparatus and rough endoplasmic reticulum indicated a poor cellular condition. However, none of these morphological alterations were related to an apoptotic form of cell death. Indeed, during our TEM investigation, no nuclear alterations were detected, and the nucleus appeared surrounded by a continuous nuclear envelope, displayed an evident nucleolus and well-organized chromatin, and no chromatin condensation or margination was detected in the J774 cells after either glutamate or erastin treatments. Moreover, both erastin and glutamate treatment seemed to induce cell detaching and a rounded cellular shape (Figure 4E). Their cell surface appeared continuous without any signs of plasma membrane rupture or disorganization, although we did observe thin protrusions. Most of these appeared to be continuous tunneling nanotubes connecting neighboring cells (Figure 4E, asterisk) previously described as structures involved in intercellular communication [41,42].

Administration of Tβ4 in glutamate (Figure 4G,H) and erastin (Figure 4K,L) treated samples seemed to gradually reverse the alterations described above. The cytoplasmic compartment showed reorganization of its cellular structures associated with an evident diminishing of lysosomal activity. Mitochondria showed partially reconstituted cristae and a continuous mitochondrial membrane. The rounded cell shape transitioned to a more flattened type.

Interestingly, in the glutamate and erastin-treated cells, we frequently detected the presence of tunneling nanotubes (TNT) connecting neighboring cells (Figure 4E, asterisk). TNT are considered dynamic structures whose formation and organization can be affected by different pathological conditions. It has been reported that oxidative stress may induce TNT formation in astrocytes and hippocampal neurons [43]. Inflammation and even viral infections have been observed to spread via tunneling nanotubes. They may also be involved in the cell-to-cell transfer of signal transduction molecules. Fas ligand (FasL) involved in programmed cell death has been demonstrated to stimulate TNT formation, promoting the propagation of cell death signals to neighboring cells [44]. Our results suggest a possible involvement of the ferroptotic process in the cell surface reorganization and TNT formation, which may facilitate diffusion of the ferroptosis process between connected cells. Considering that tunneling nanotubes are F-actin-containing protrusions of the cell surface, Tβ4 could modulate ferroptosis not only by its metal binding sites, as described above, but also through its G-actin binding properties. Tβ4, as a major G-actin sequestering protein, could prevent tunnelling nanotube formation in erastin and glutamate treated J774 cells inhibiting propagation of ferroptotic cell death signals to the connected cells.

These morphological results clearly show that glutamate and erastin induce ferroptosis in J774 cells, while Tβ4 administration after 4 h can inhibit the process and reverse the critical cellular condition.

**Figure 5:**
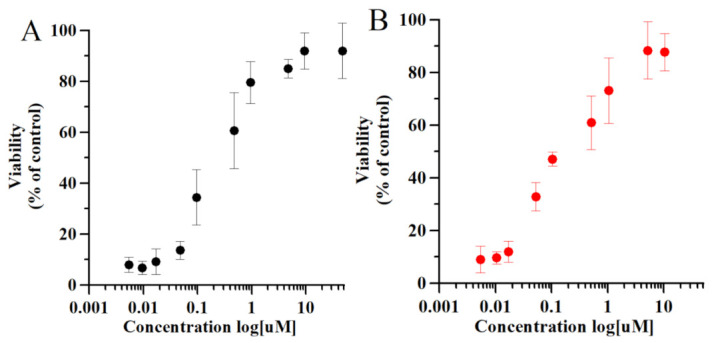
Dose-response relationship for inhibition of erastin-induced (10 μM, 24 h) death in J774 cells by Fer-1 (**A**) and Tβ4 (**B**). Cell viability quantitative data are shown as mean and standard error. Statistical comparison of the cell viability quantitative data in different experimental conditions were performed, with 5.0 GraphPad Prism software, by the unpaired t-test. The data were also analyzed with one-way ANOVA. *p* values < 0.05 was considered significant.

In the next experiment, we examined the inhibitory activity of Tβ4 and compared it with the activity of Ferrostatin-1 (Fer-1), a known ferroptosis (erastin-induced) inhibitor previously described by Dixon et al. [3]. The activity of both inhibitors was measured by the cell viability assay (Figure 5) in the cell cultures incubated with erastin and increasing concentration of inhibitors. The dose-response effect of Tβ4 (Figure 5A) can be compared with the effect of Fer-1 in the same experimental conditions.

Fer-1 is a synthetic compound isolated by high-throughput screening of small molecule libraries as a potent inhibitor of ferroptosis [3]. Fer-1 prevents erastin-induced accumulation of cytosolic and lipid ROS [3]. Recent electrochemical studies showed that Fer-1 forms a complex with iron ions, supporting the theory that the anti-ferroptotic activity of Fer-1 is actually due to the scavenging of initiating alkoxyl radicals produced by ferrous iron from lipid hydroperoxides [45]. Indeed, Fer-1 derivatives substituting both amine nitrogen atoms, thus iron-binding sites, lack inhibitory activity [3]. The same inhibitory results (Figure 5), obtained for Fer-1 and Tβ4, suggest the same inhibitory mechanism through complex formation with iron ions. In addition, numerous studies showed that inhibitory activity of Fer-1 is correlated with the gene regulation of proteins involved in cellular stress processes [46]. For this reason, we examined the gene expression of selected proteins involved in stress-oxidative processes. In the next experiments, we focused on the inhibitory mechanisms of Tβ4 rather than Fer-1, while the activity of the latter is described elsewhere.

According to Dixon [1], ferroptosis could be defined as a sabotage mechanism where the cell inhibits its own normal processes. However, ferroptosis is related to oxidative stress, and consequently, the involvement of oxidative stress proteins is expected. Indeed, among thirty different genes studied in relation to ferroptosis [47], many are involved in oxidative stress. In our study, we chose four genes, namely BAX (Bcl-2 associated X-protein), HSP-70 (heat shock protein 07), which are involved in the oxidation process, but are poorly described in connection with ferroptosis; and HO-1 (hem oxygenase) and TXNRD-1 (Thioredoxin reductase 1) which were recently linked to ferroptosis [46].

**Scheme 2:**
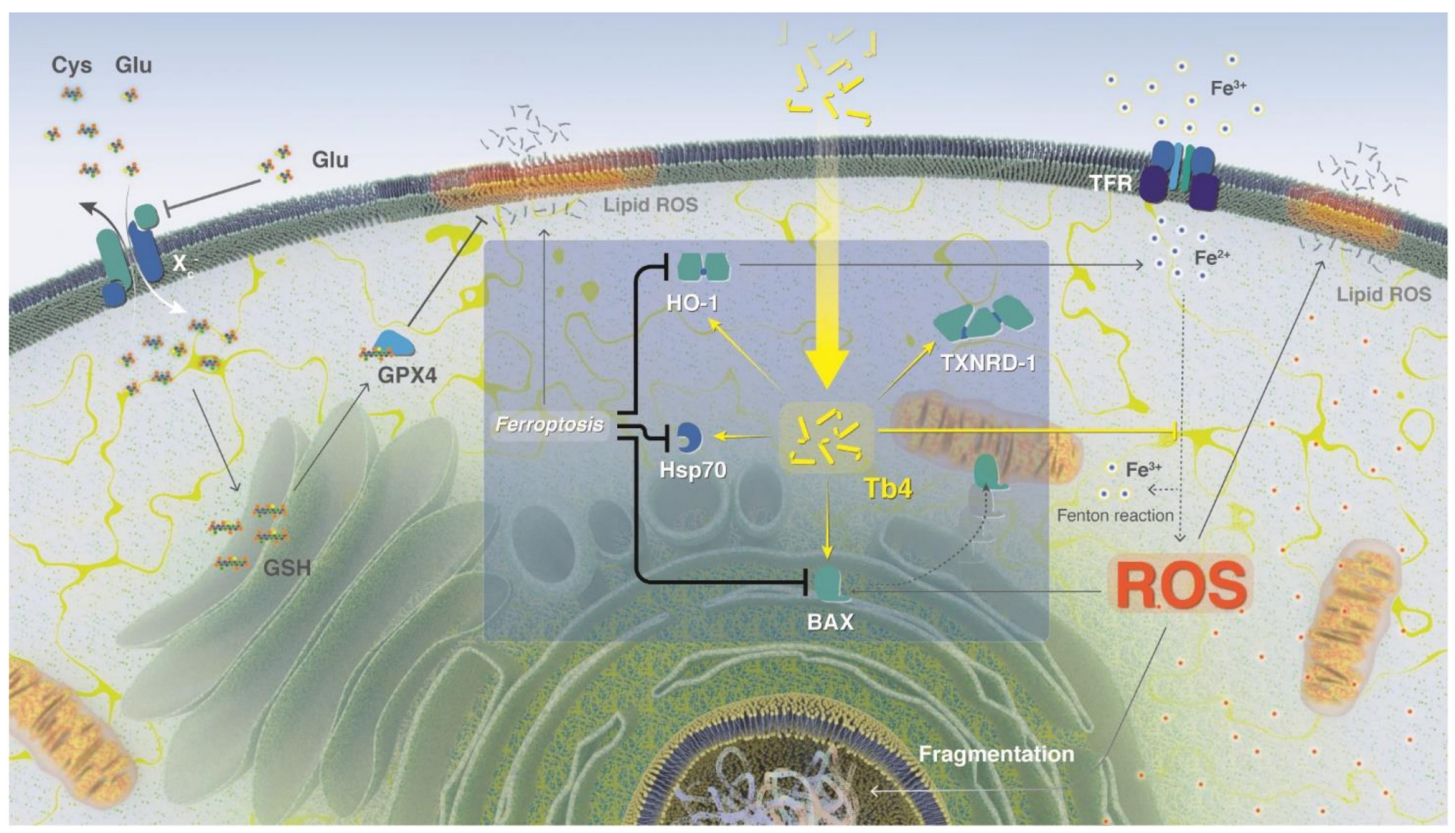
Tβ4 inhibitory activity of ferroptosis. An excess of L-Glu in the extracellular matrix leads to the inhibition of GSH synthesis and reduced anti-oxidative activity, which increases the concentration of free iron ions in the cell and enhances the production of reactive oxygen species (ROS). High ROS and free iron ion concentrations trigger ferroptosis. Ferroptosis leads to lipid membrane disruption by ROS, and silences the expression of HO-1, Hsp70, and BAX. Extracellular treatment with Tβ4 can inhibit ferroptosis by increasing the expression of HO-1, Hsp70, BAX, and TXNRD-1, and direct chelation of free iron ions. The scheme was created by Heno Hwang, scientific illustrator at King Abdullah University of Science and Technology (KAUST). This image is original and designed specifically for the targeted publication. It should not be cropped, distorted, or in any way edited without the expressed consent of Research Publication Services.

Bax is a cytosol protein, which translocates into mitochondria when apoptosis occurs [48] (Scheme 2). Jungas et al. [49] studied glutamate toxicity and regulation in neuronal development. Their studies showed BAX-activation mechanism dependent on glutathione, with the downstream effect of glutathione depletion leading to the direct activation of BAX. Moreover, Bax and its transcript increase significantly in cerebral ischemia-injured neurons [50,51], suggesting that glutamate could be involved in the mechanism of BAX expression control. Of note, Tβ4 reduces the intracellular ROS levels induced by H_2_O_2_, decreases Bax/BCl_2_ ratio, and increases the expression of antiapoptotic proteins in cardiomyocytes [52] and cardiac fibroblasts [53].

Heat shock protein 70 (Hsp70) is a stress-responsive protein increasing under stress conditions, namely heat, hypoxia, and glucose deprivation. Increased Hsp70 concentration leads to higher stress tolerance and cytoprotection [54]. Hsp70–mediated cytoprotective effects were studied in vitro in numerous cell/tissue types under changing environmental conditions of stress. Noteworthily, Hsp70 has significant cytoprotective effects under ischemic conditions. For instance, induction of Hsp70 (e.g., by enforced overexpression with viral vectors or drugs) protects brain cells against ischemic injuries [55,56,57,58]. Oxidative stress and antioxidants can influence Hsp70 expression [59,60], while the reduction in Hsp70 expression leads to higher ROS generation and mitochondrial protein oxidation [61]. Guo et al. [54] showed that Hsp70 regulates cellular redox status by changing the activities of the GSH-related enzymes, glutathione peroxidase, and glutathione reductase, in response to hypoxic and ischemic stress. The switching of antioxidant enzyme activities could be a critical mechanism mediating the enhanced cytoprotection afforded by the enhanced expression of Hsp70. Despite numerous studies and different theories, the exact molecular mechanisms of Hsp70 action in ischemic conditions remains undefined

Heme oxygenase (HO)-1 metabolizes heme into biliverdin/bilirubin, carbon monoxide, and ferrous iron, and has been suggested to demonstrate cytoprotective effects under various stress-related conditions. HO-1 is commonly regarded as a survival molecule, exerting an important role in cancer progression, and its inhibition is considered beneficial in several cancers [62]. HO-1 is often upregulated in tumor tissues, and its expression is further increased in response to therapies [63]. The role of HO-1 in ferroptosis is extensively described in the recent review [62]. Depletion of cellular glutathione has been shown to increase HO-1 gene transcription in the mouse motor neuron-like hybrid cells, NSC34 cells [64]. The correlation between HO-1 expression and ferroptosis is unclear. In HT-1080 fibrosarcoma cells, Erastin induces a time- and dose-dependent increase of HO-1 expression [65]. However, HO-1 also functions as a negative regulator in erastin- and sorafenib-induced hepatocellular carcinoma, since knockdown of HO-1 expression enhanced cell growth inhibition by erastin and sorafenib [66].

The selenoprotein thioredoxin reductase 1 (TXNRD1 gene) is a cytoplasmic protein that decreases thioredoxin levels and protects against oxidative stress [67]. Recently, it was shown that TXNRD1 is a strong negative modulator of ferroptosis susceptibility in pancreatic cancer cells [68]. Moreover, a high-dose (25 mg/kg) of the anti-rheumatoid arthritis drug auranofin (AUR) upregulates hepcidin expression and induces ferroptosis, and causes lipid peroxidation through inhibition of thioredoxin reductase (TXNRD) activity, in C57BL/6J mice and a mouse model of hemochromatosis (Hfe−/− mice) [69].

**Figure 6:**
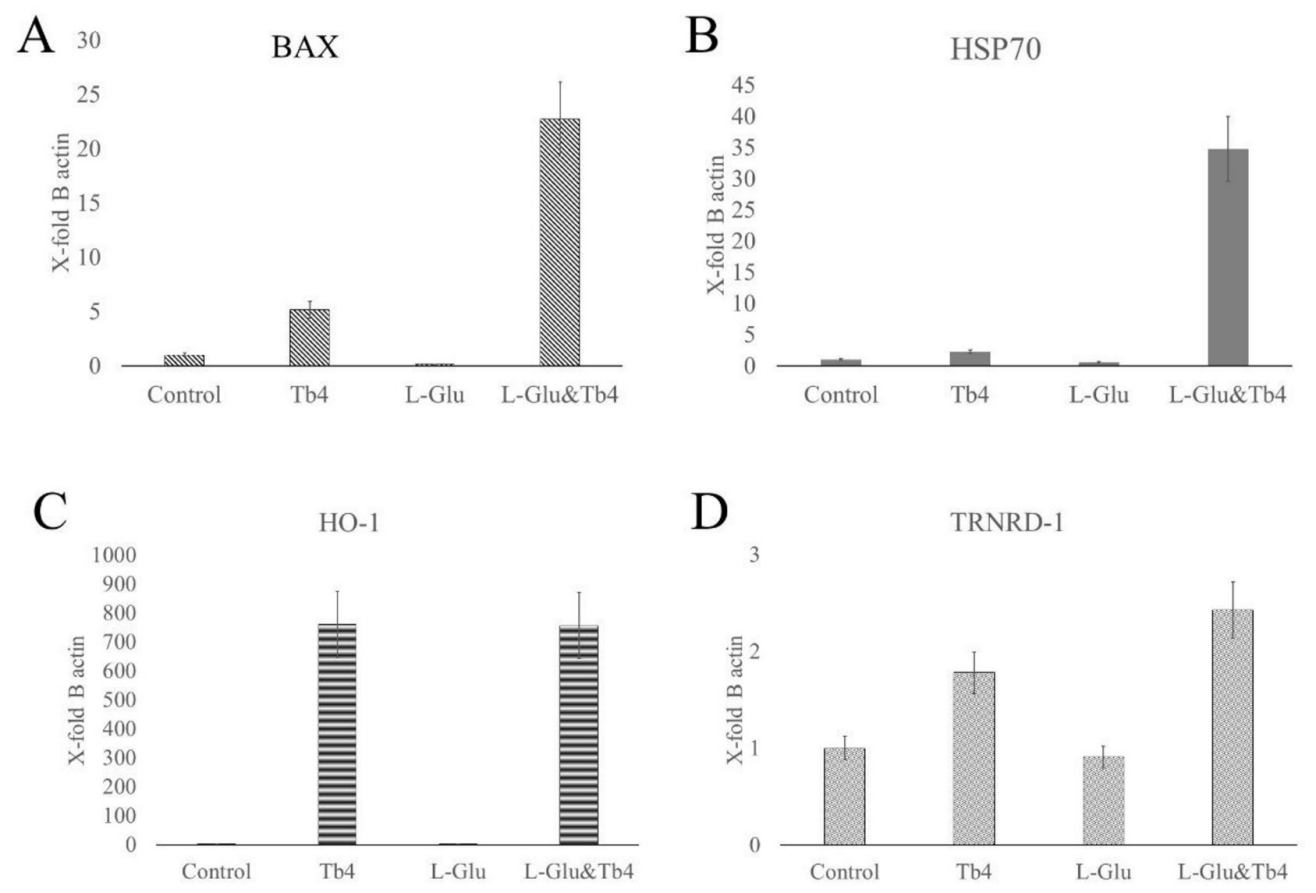
mRNA expression levels (mean and standard error) of (**A**) BAX, (**B**) HSP70 (**C**) HO-1, and (**D**) TRNRD-1, which are related to stress conditions, in J774 cells. The amount of mRNA of each gene for each experiment was normalized with the respective value of the beta-actin housekeeping gene mRNA (X-fold of beta-actin expression). Statistical comparison of the gene’s expression in different experimental conditions was performed with 5.0 GraphPad Prism software. The data were also analyzed with one-way ANOVA. *p* values < 0.05 was considered significant.

Here, we evaluated the levels of BAX, HO-1, Hsp70, and TRNRD-1 mRNAs (Figure 6) under normal and ferroptosis conditions, which were induced by the extracellular overload of glutamate. In addition, we evaluated mRNA expression levels of these proteins in the presence of high extracellular concentrations of thymosin. Finally, we examined their mRNA levels in the cells treated with thymosin after 4 h of ferroptosis induction. We observed that glutamate overload led to the downregulation of all studied genes; conversely, the presence of Tβ4 upregulated mRNA expression, particularly of HO-1. Notably, the downregulation of glutamate could be inverted by the successive treatment with Tβ4.

## 3. Discussion

Thymosin β4 is highly conserved across the animal kingdom, from amphibians to mammals, and its amino acid sequence is well conserved [15]. Among mammalian species, the amino acid sequences of human, rat, murine, bovine, porcine, and ovine Tβ4 are identical [70]. Thymosin β4 is encoded by the TMSB4X gene. Mouse, rat, zebrafish, frog, and pig display differential mRNA expression of TMSB4X in organs, while in humans, it is widely distributed in tissues with high concentrations of different essential metal ions [71] (Scheme S1).

Iron is an essential metal ion with a fundamental role in diverse cellular processes, such as DNA synthesis, proliferation, cell cycle regulation, and the function of proteins containing iron–sulfur clusters [72]. Iron–sulfur cluster-containing proteins include enzymes that contribute to maintaining genomic stability, as well as respiratory function [72]. Indeed, rapidly reproducing cancer cells re-program iron metabolism in ways that result in net iron influx [8,72]. Iron is a highly reactive metal due to its redox potential, and when present in a free ion form, it contributes to the production of reactive oxygen species (ROS) in the Fenton reaction (Scheme 2). Oxidative and iron-dependent cell death with neurodegenerative consequences was previously described in epilepsy, stroke, and other trauma situations [73,74,75]. Importantly, increased levels of iron were found in the central nervous system of patients with neurodegeneration [5]. Surprisingly, elevated iron concentrations in cancer cells do not lead to cell disruption, but the exact mechanism of cell defense remains unknown. Cancer cells have developed many different iron-regulating mechanisms, which permit them to benefit from high iron concentrations without suffering from the harmful effects of free radicals. Many iron-regulating processes in tumor pathology have already been discovered [76], while others are unknown. Considering the iron-dependent etiology of ferroptosis, it is likely that some of the unknown iron-defense processes in tumor cells can inhibit ferroptosis-induced cell death.

The involvement of Tβ4 in different cancer types has been widely described in recent years (Table S4). It is noteworthy that among the 13 most common cancer types (https://www.healthline.com/health/most-common-cancers, accessed date: 1 February 2021), the nine tissues with the highest cancer incidence also have high thymosin mRNA expression (Scheme S1, Table S4), which is further enhanced in tumors (Table S4). Recently, it was shown that ferroptosis is fundamental in anticancer therapy, and efficient anticancer drugs can induce ferroptosis-cell death (Table S4).

Neurodegeneration is characterized by neuronal cell death. Detailed biochemical and morphological studies of neuronal tissues have linked neurodegeneration with increased iron concentrations in the tissue and successive production of free radicals [77,78]. Since ferroptosis was first described in 2012 [3], numerous research data have correlated neurodegenerative processes with iron-ROS induced cell death (Table S4) and glutamate-induced cell injury [79]. Up to now, Tβ4 has never been linked to ferroptosis, but different experimental studies have shown the beneficial effects of Tβ4 in neurodegeneration (Table S4).

Ferroptosis can be induced in vivo in rat hippocampal slices and primary oligodendrocyte models as well as in ischemia-reperfusion injury models, by high concentrations of extracellular iron or glutamate, or by depletion of extracellular cysteine. In vitro, ferroptosis can be induced by physiological conditions (e.g., high extracellular iron or glutamate) or small molecules (e.g., erastin), which blocks system X_c_^−^-mediated Cys_2_ import [1] (heterodimer composed of solute carrier family 7, member 11 (SLC7A11) and SLC3A2; Scheme 2), while glutamate receptors are not involved in ferroptosis [80]. Erastin and glutamate are termed type I ferroptosis inducers that inhibit cysteine uptake by the cysteine/glutamate antiporter (system x_c_^−^), enabling glutathione synthesis (Scheme 2), and thereby lowering antioxidant defenses [3].

Glutamate-induced oxidative stress leads to lipid peroxidation-mediated ferroptosis (Scheme 2) and is a major contributor to neurodegenerative pathologies [81]. In recent studies, L-Glutamate (3 h, 5 mM) mimicked in vitro the consequences of stroke and neurodegenerative pathologies in cell cultures [82,83]. The addition of iron enhances glutamate-induced cell death [81], while inhibition of ferroptosis prevents glutamate-induced cell death in organotypic hippocampal slice cultures [3]. Importantly, glutamate activity can be inhibited by iron chelators (e.g., Desferal) [3].

Ferroptosis, in contrast to other RCDs processes can be inhibited, thus inhibiting molecules, namely iron chelators and lipophilic antioxidants, that could be used as a molecular switcher of the entire process. Our metal coordination studies showed that Tβ4 could bind both Fe^2+^ and Fe^3+^ metal ions in vitro in four different metal binging regions (Figure 3). Iron chelating ability of Tβ4 takes a part of iron cellular homeostasis, and increased concentration of Tβ4 leads to upregulation of HO-1 protein expression (Figure 6B), which metabolizes heme into ferrous iron.

Tβ4 as endogenous iron chelator influences anti-oxidative processes related to free iron ions and ROS production in Fenton reaction. Recently, Zhu et al. [84] showed that Tβ4 reduces oxidative stress and lipid peroxidation in the injured liver model in mice by inhibiting ferroptosis via up-regulation of GPX4 expression. In our experimental conditions, increased concentration of Tβ4 enhances the expression of oxidative stress-related genes: BAX, HSP70, and TRNRD-1, which are downregulated during ferroptosis. Noteworthily, the expression of these genes is even higher when Tβ4 concentration increases during the ferroptosis process (Figure 6A,D). Such observation suggests that Tβ4 expression and/or supplementation could be used to enhance anti-oxidative processes and further inhibit ferroptosis.

In our experimental conditions, erastin- and glutamate-induced ferroptosis leads to different ultrastructural alternations of cell morphology (Figure 4E,F,I,J). Some of them, e.g., those involving mitochondria, were previously described in ferroptosis processes, while the others—disruption of Golgi apparatus and rough endoplasmic reticulum, continuous tunneling nanotubes connecting neighboring cells—are less investigated in this context and need further studies. Importantly, Tβ4 administration during ferroptosis can gradually reverse the morphological alternations (Figure 4G,H,K,L).

## 4. Conclusions

Thymosin β4 is an iron chelator, which takes part in the iron homeostasis mechanism and iron-related oxidative stress. Administration of Tβ4 enhances the expression of oxidative stress genes (HO-1, BAX, HSP70, and TRNRD-1), which are suppressed by glutamate-induced ferroptosis. The characteristic morphological alternations of the ferroptosis process (e.g., predominant mitochondrial shrinking, an electron-dense ultrastructure, reduced/disappeared cristae, and ruptured outer mitochondrial membrane) can be reversed by the administration of Tβ4, which restores normal cytoplasmic compartment organization. In this context, Tβ4 can be considered a ferroptosis switcher. A better understanding of Tβ4 pathological functioning in cancer cells, and its beneficial effects in neurodegeneration, will enable us to switch different pathological states into physiological processes.

## 5. Experimental Procedures

### 5.1. Reagents

Human Thymosin β4 (HPLC > 98%) was purchased from Regene RX, Rockville, MA, USA. Erastin, L-glutamate, BODIPY-C11, formaldehyde, glutaraldehyde in 0.1 M sodium cacodylate buffer pH 7.4, osmium tetroxide, uranyl acetate, ethanol, and xylene were Sigma Aldrich products; purity 99%. All high-purity grade reagents were used without further purification.

### 5.2. EPR Experimental Details

All EPR spectra were recorded using a continuous wave Bruker ELEXSYS E500 electron paramagnetic resonance (EPR) spectrometer operating at the X band equipped with a superhigh Q (ER 4122 SHQ) resonator. To create comparable data, the spectra were measured using the same conditions with instrumental parameters as described previously [85,86]. Bruker Xenon software (Bruker BioSpin, Rheinstetten, Germany) was used for both data collection and spectral processing.

### 5.3. NMR

NMR samples were prepared by dissolving 1.0 mg of Tβ4 in 500 μL of D_2_O. The pD was adjusted by adding DCl or NaOD and calculated as pD = pH (pH meter reading) + 0.4 [87]. All NMR experiments were performed on 800 MHz spectrometers equipped with a triple resonance TCI probe and Z gradients coils. The temperature was kept at 25 °C and it was calibrated with an external standard mixture of ethylene glycol and DMSO-D_6_ by monitoring the chemical shift difference between the hydroxyl and methylene proton. The chemical shifts were calibrated with respect to standard DSS for proton, and ^13^C chemical shifts were calibrated indirectly. D_2_O was used for locking.

Apart from the one-dimensional spectrum with water suppression using excitation sculpting, two-dimensional homonuclear TOCSY, NOESY, hetero-nuclear HSQC and HSQC-TOCSY (with mixing 16 ms) experiments were recorded. For the heteronuclear experiments, 2048 and 360 data points were collected in proton and carbon dimensions, respectively, which corresponds to 125 ms and 17 ms acquisition time in the respective dimension. The data were apodized with sine function and zero filled with 4096 and 1024 points in proton and carbon dimensions, respectively. The recycle delay was kept for 1 and 1.5 s, respectively, for HSQC and TOCSY-HSQC. The total measurement time was 300 and 240 min for HSQC and TOCSY-HSQC, respectively. The sample was stable during measurement.

Five different samples were prepared with the addition of individually FeCl_2_ and FeCl_3_ with thymosin: metal ion ratios 1:0.05, 1:0.1, 1:0.2, 1:0.5, and 1:1; and five different samples were prepared with the addition of AlCl_3_ with Tβ4: metal ion ratios 1:0, 1:1, 1:2, 1:5 and 1:10. For the sample with some AlCl_3_, slightly visible precipitation was noticed.

The diffusion was measured using the pulsed field gradient (PFG) NMR on a Bruker Avance 800 MHz spectrometer equipped with a triple resonance TCI probe and Z gradients coils. The pulse sequence incorporates longitudinal eddy–current delay (LED) along with bipolar gradients. All the diffusion measurements were recorded in a pseudo-two-dimensional manner. In the process, we acquired 24 spectra over the gradient strengths ranging from 2% to 95% with a linear variation of the gradient strength. The diffusion time was kept 175 ms along with 32 scans and 1.5 s recycle delay. The decaying signal intensity was analyzed using the following equation,

I = I_0_ * exp{−Dγ^2^g^2^δ^2^(Δ − δ/3)}

I is the observed intensity, I_0_ is the reference intensity, D is the diffusion coefficient, γ is the gyromagnetic ratio of the observed nucleus, g is the gradient strength, δ is the length of the gradient, and ∆ is the diffusion time.

All NMR data were processed using Topspin 4.0.7 and analyzed with Sparky software. Nearly complete assignments of the ^1^H and ^13^C resonances for all the residues were carried out by application of a standard and well-established procedure using HSQC and TOCSY-HSQC [88]. We also used the PROSECCO (PROtein SEquences and Chemical shift Correlations) server, which predicts the chemical shift of both folded and unfolded proteins, which is sequence-based. Since Tβ4 is intrinsically disordered in D_2_O, we used the server accordingly. The earlier assignment of the apo form of Tβ4 was transferable.

### 5.4. DFT

#### System Setup and Conformational Analysis

Conformational analyses were carried out on the full-length N-acetylated thymosin β-4. The protein sequence was built with Maestro v. 12.5 as a random coil (no secondary structure elements) amino acid sequence, as this is the most likely Tβ4 structure in solution [22]. An acetyl group is added to the N-terminal serine residue, whereas the C-terminal serine residue is taken in its charged COO^−^ state, in agreement with the physiological form of Tβ4 [22]. Therefore, the initial system is composed of the random coil protein and one Fe^3+^ metal ion placed nearby the protein. The system was modeled with the OPLS-AA force field [89] as implemented in the MacroModel v12.4 package. Choice of the metal ion is dictated by the absence of Al^3+^ parameters in this force field. The MacroModel v12.4 [90] module was used along with the MonteCarlo Multiple Minimum (MCMM) algorithm [91]. Three different conformational analyses were performed; in the first one, the number of search iterations was set to 50,000 steps and the Polak–Ribiere conjugate gradient (PRCG) minimization algorithm was used [92] set to 10,000 minimization steps and the gradient of first derivatives convergence threshold set to 0.005. The second and third conformational analyses were run for a total of 10,000 search steps using truncated-Newton conjugate gradient minimization algorithm (TNCG) [93], with 20,000 minimization steps and the gradient of second derivatives convergence threshold set to 0.05. All torsional variables were turned on during the conformational search, with a maximum atom deviation cutoff of 0.8 Å to discriminate between redundant conformers. The energy window for saving structures was set to 20.0 kcal/mol.

Through visual inspection of all the conformers produced by the three conformational runs, we selected 11 structures whose metal-binding mode lies in one of the four metal anchoring areas predicted by NMR and CSP measurements (Figure 1). Three binding modes were identified: Tβ4^N-N^, in which the metal was bound to the N-terminal of the protein (ASP^2^, ASP^5^, GLU^8^ and GLU^10^ residues); Tβ4^N-mid^ in which the metal was bound to the N-terminal and the middle of the protein (ASP^2^, ASP^5^, GLU^10^ and GLU^24^ residues); Tβ4^N-C^ in which the metal was bound to the N-terminal and the C-terminal regions of the protein (ASP^5^, GLU^8^, GLU^21^ and GLU^35^ residues).

Five conformers for the Tβ4^N-C^ and Tβ4^N-mid^ binding modes were chosen, and one for the Tβ4^N-N^ binding mode, for a total of eleven initial structures.

### 5.5. Molecular Dynamics Simulations

The eleven Fe^3+^-Tβ4 structures selected from the previous conformational analyses were immersed in a dodecahedron box with the solute kept to a distance of 20 Å from the box and solvated with ≃ 8000 TIP4P-ew [94] water molecules along with a salt concentration of 150 mM NaCl. The protein was modeled with the AMBER99SB*-ILDN-Q force field [95,96], suitable for intrinsically disordered proteins. Although conformational analyses have been performed with Fe^3+^ ion, MD simulations were performed using the Al^3+^ metal ion. This choice is due to the fact iron is a paramagnetic element, therefore, NMR and CSP data for Al(III) (Table 1) are more reliable. Parameters for the Al^3+^ ion were taken from Pengfei Li et al. [97] as they have been developed for the AMBER family of force fields and the TIP4P-ew water model.

Long-range electrostatic interactions were evaluated using a particle-mesh Ewald (PME) approach [98], and Lennard-Jones interactions were truncated at 12~Å with an atom-based force switching function which is effective at 10 Å. After 5000 steps of steepest descent minimization, the system was equilibrated in a multistep manner with decreasing force constants according to the scheme illustrated in Table S3. The production run was conducted in the NPT ensemble for 1 μs for each run. All atoms were constrained with the LINCS algorithm [99]; hydrogen atoms were treated as virtual interaction sites so that to allow a time step of 4.0 fs while maintaining energy conservation [100]. Simulations were performed at the temperature of 310 K and 1.0 atm pressure. The V-rescale thermostat (JCP 126, 014101) with a time constant of 0.1 ps was used to control the temperature. The isotropic Berendsen barostat was used for the equilibration stage, whereas the Parrinello-Rahman one [101] was used for production runs; the reference pressure was set to 1.0 atm, the time constant to 2.0 ps and the isothermal compressibility to 4.5 × 10^−5^ in order to maintain the pressure of the system. Dispersion correction was used to correct long range dispersion of energy and pressure.

### 5.6. Cell Cultures

Commercial cell line J774 (ICLC ATL98011) was obtained from the Istituto Nazionale per la Ricerca sul Cancro c/o CBA (ICLC, Genova). The culture medium used for this purpose was a mixture of MEM (EBSS), 10% fetal bovine serum (FBS), 100 units/mL penicillin, 100 mg/mL streptomycin, 2 mM L-Glutamine, 1% non-essential amino acids. To perform different experimental conditions, confluent cells were isolated using trypsin/EDTA and, for the experimental procedure, samples of 2–3 × 10^4^ cells/cm^2^ J774 cells were plated on different glass coverslips at 37 °C, 5% CO_2_. The daily prepared water solution of Tβ4 was added to the cell growing medium to the final 10 μM concentration.

### 5.7. Cell Viability Measurements

The cell viability was evaluated using the Luna^FL^ Cell Counter (www.logosbio.com, accessed date: 2 March 2021) as indicated in the manufacturer’s user manual. Cell viability under test conditions is reported as a percentage relative to the negative control treatment.

For each of three analyses (repeated experiments), three distinct biological replicas were made, and quantitative data were expressed as a mean. Cell viability quantitative data were expressed as mean + standard error. Statistical comparison of the cell viability quantitative data in different experimental conditions were performed, with 5.0 GraphPad Prism software, by the unpaired t-test. The data were also analysed with one-way ANOVA. *p* values < 0.05 was considered significant.

### 5.8. Gene Expression

The expression of the following genes at the mRNA level was evaluated by real-time PCR: BAX; HSP70; HO-1 and TXNRD-1. The cells were immediately frozen after treatment and stored at −80 °C until RNA extraction. The RNA was extracted with NorDiag ARROW RNA kit using the automated tool of the same company. Actin was used as a reference gene [102]. The Real-time reverse-transcriptase PCR was performed using the Roche Light Cycler system and SYBR Green I kit amplification kit (Roche Diagnostics). The following primers (b-actF 1⁄4 50-GCATGGGTCAGAAGG-30, b-actR 1⁄4 50-AGGCGTA-CAGGGATAG-30) were designed using the sequences of the human beta-actin mRNA (Gen-Bank accession no. NM_001101).

Real time was performed in 20 μL of final volume containing: 3 mM MgCl_2_, 0.25 mM of each primer, and 2 μL of RNA extract. Cycling was performed using the following amplification conditions: an initial reverse transcription at 55 °C for 10 min, denaturation at 95 °C for 30 s followed by 35 cycles at 95 °C for 10 s, 53 °C for 10 s and 72 °C for 8 s with subsequent melting analysis: heating to 95 °C for 20 s, cooling to 5 °C for 10 s and re-heating to 95 °C per second.

Fluorescence was detected at the end of the 81 °C segment in the PCR step (single mode) and at 45 °C segments in the melting step (continuous mode) in the F1 channel. The relative gene expression was analyzed by using the 2-DDCT method [103]. For each of three analyses (repeated experiments), three distinct biological replicas were made, and quantitative data were expressed as a mean. Folding change values in genes expression relative to the beta-actin have been represented as mean + standard error. Statistical comparison of the gene’s expression in different experimental conditions was performed with 5.0 GraphPad Prism software. The data were also analyzed with one-way ANOVA. *p* values < 0.05 was considered significant.

### 5.9. TEM Microscopy

Samples of J774 cells used in this study were fixed for 2 h in a solution of 1% (para)formaldehyde and 1.25% glutaraldehyde in 0.1 M sodium cacodylate buffer, pH 7.4. They were post-fixed in 1% osmium tetroxide for 1 h and stained in aqueous uranyl acetate 0.25% overnight at 4 °C. Cell cultures underwent a dehydration process in an ascending graded series of ethanol and xylene, followed by infiltration and embedding in Embed 812 resin. The specimens were transferred to flat embedding molds and polymerized in the oven at 60 °C for 24 h. 60–90 nm thin sections were cut with an LKB ultratome 8800 ultramicrotome, post-stained with uranyl acetate and bismuth subnitrate, observed and photographed using a transmission electron microscope (JEOL 1400 plus model, Tokyo, Japan) operating at 80 kV.

## Figures and Tables

**Table 1:** Amino acid residues affected by metal/peptide interactions. Metal-binding regions are also signed in Scheme 1.

Binding Region	I							II					III					IV					
Fe (II)	S1	K3	P4	-	M6	-	-	K11	F12	D13	S15	L17	T22	P27	L28	P29	S30	T33	I34	-	-	A40	S43
Fe (III)	S1	K3	P4	-	M6	I9	-	-	F12	D13	S15	-	T22	-	L28	-	-	T33	I34	Q35	E36	A40	-
Al (III)	-	-	P4	D5	M6	I9	E10	-	F12	D13	-	-	-	-	L28	-	-	-	I34	Q35	E36	-	-

**Table 2:** Percentage of hydrogen bond and salt bridge occupancies during the simulations. bb: backbone, sc: side chain.

TB4^N-C^			TB4^N-mid^			TB4^N-N^		
Donor	Acceptor	Occ. (%)	Donor	Acceptor	Occ. (%)	Donor	Acceptor	Occ. (%)
GLU^35^-N_bb_	THR^33^-O_sc_	27.6	LYS^38^-N_bb_	GLU^35^-O_bb_	26.0	THR^22^-N_bb_	LYS^19^-O_bb_	23.8
LEU^17^-N_bb_	LYS^14^-O_bb_	25.8	LEU^28^-N_bb_	ASN^26^-O_sc_	28.2	GLN^23^-N_bb_	LYS^18^-O_bb_	22.2
LEU^28^-N_bb_	ASN^26^-O_sc_	36.6	ILE^34^-N_bb_	LYS^31^-O_bb_	26.7	GLU^21^-N_bb_	LEU^17^-O_bb_	23.4
THR^33^-N_bb_	GLN^36^-O_bb_	32.0	LYS^31^-N_bb_	LEU^28^-O_bb_	21.1	ILE^9^-N_bb_	MET^6^-O_bb_	53.0
LYS^31^-N_bb_	LEU^28^-O_bb_	22.5	_-_	_-_	-	GLU^8^-N_bb_	ASP^5^-O_bb_	51.5
-	-	-	-	-	-	LYS^38^-N_bb_	GLU^35^-O_bb_	20.0

## Data Availability

Not applicable.

## Supplementary Materials

The following supporting information can be downloaded at: https://www.mdpi.com/article/10.3390/ijms23010551/s1.

## References

[1] Dixon S.J. (2017). Ferroptosis: Bug or feature?. Immunol. Rev..

[2] Galluzzi L., Vitale I., Aaronson S.A., Abrams J.M., Adam D., Agostinis P., Alnemri E.S., Altucci L., Amelio I., Andrews D.W. (2018). Molecular mechanisms of cell death: Recommendations of the Nomenclature Committee on Cell Death 2018. Cell Death Differ..

[3] Dixon S.J., Lemberg K.M., Lamprecht M.R., Skouta R., Zaitsev E.M., Gleason C.E., Patel D.N., Bauer A.J., Cantley A.M., Yang W.S. (2012). Ferroptosis: An Iron-Dependent Form of Nonapoptotic Cell Death. Cell.

[4] Yagoda N., von Rechenberg M., Zaganjor E., Bauer A.J., Yang W.S., Fridman D.J., Wolpaw A.J., Smukste I., Peltier J.M., Boniface J.J. (2007). RAS–RAF–MEK-dependent oxidative cell death involving voltage-dependent anion channels. Nature.

[5] Ndayisaba A., Kaindlstorfer C., Wenning G.K. (2019). Iron in neurodegeneration–cause or consequence?. Front. Neurosci..

[6] Carbonell T., Rama R. (2007). Iron, oxidative stress and early neurological deterioration in ischemic stroke. Curr. Med. Chem..

[7] Wang H., An P., Xie E., Wu Q., Fang X., Gao H., Zhang Z., Li Y., Wang X., Zhang J. (2017). Characterization of ferroptosis in murine models of hemochromatosis. Hepatology.

[8] Bogdan A.R., Miyazawa M., Hashimoto K., Tsuji Y. (2015). Regulators of Iron Homeostasis: New Players in Metabolism, Cell Death, and Disease. Trends Biochem. Sci..

[9] Cassimeris L., Safer D., Nachmias V.T., Zigmond S.H. (1992). Thymosin beta 4 sequesters the majority of G-actin in resting human polymorphonuclear leukocytes. J. Cell Biol..

[10] Philp D., Kleinman H.K. (2010). Animal studies with thymosin β4, a multifunctional tissue repair and regeneration peptide. Ann. N. Y. Acad. Sci..

[11] Smart N., Rossdeutsch A., Riley P.R. (2007). Thymosin β4 and angiogenesis: Modes of action and therapeutic potential. Angiogenesis.

[12] Faa G., Piras M., Mancuso L., Coni P., Pichiri G., Orrù G., Fanni D., Gerosa C., Cao G., Taibi R. (2021). Thymosin beta-4 prenatal administration improves fetal development and halts side effects due to preterm delivery. Curr. Med. Chem..

[13] Goldstein A.L. (2003). Thymosin β4: A New Molecular Target for Antitumor Strategies.

[14] Pardon M.-C. (2018). Anti-inflammatory potential of thymosin β4 in the central nervous system: Implications for progressive neu-rodegenerative diseases. Expert Opin. Biol. Ther..

[15] Erickson-Viitanen S., Ruggieri S., Natalini P., Horecker B. (1983). Distribution of thymosin β4 in vertebrate classes. Arch. Biochem. Biophys..

[16] Huff T., Rosorius O., Otto A.M., Müller C.S.G., Ballweber E., Hannappel E., Mannherz H.G. (2004). Nuclear localisation of the G-actin sequestering peptide thymosin β4. J. Cell Sci..

[17] Piludu M., Piras M., Pichiri G., Coni P., Orrù G., Cabras T., Messana I., Faa G., Castagnola M. (2015). Thymosin Beta 4 May Translocate from the Cytoplasm in to the Nucleus in HepG2 Cells following Serum Starvation. An Ultrastructural Study. PLoS ONE.

[18] Zhang Y., Feurino L.W., Zhai Q., Wang H., Fisher W.E., Chen C., Yao Q., Li M. (2008). Thymosin beta 4 is overexpressed in human pancreatic cancer cells and stimulates proinflammatory cytokine secretion and JNK activation. Cancer Biol. Ther..

[19] Lee S.Y., Park M.J., Lee H.K., Son H.J., Kim C.N., Kim J.H., Kang D.W. (2017). Increased expression of thymosin β4 is in-dependently correlated with hypoxia inducible factor-1α (HIF-1α) and worse clinical outcome in human colorectal cancer. J. Pathol. Transl. Med..

[20] Wirsching H.-G., Krishnan S., Florea A.-M., Frei K., Krayenbühl N., Hasenbach K., Reifenberger G., Weller M., Tabatabai G. (2014). Thymosin beta 4 gene silencing decreases stemness and invasiveness in glioblastoma. Brain.

[21] Huang D., Wang S., Wang A., Chen X., Zhang H. (2016). Thymosin beta 4 silencing suppresses proliferation and invasion of non-small cell lung cancer cells by repressing Notch1 activation. Acta Biochim. Biophys. Sin..

[22] Lachowicz J.I., Jaremko M., Jaremko L., Pichiri G., Coni P., Piludu M. (2019). Metal coordination of thymosin β4: Chemistry and possible implications. Coord. Chem. Rev..

[23] Al-Harthi S., Lachowicz J.I., Nowakowski M.E., Jaremko M., Jaremko Ł. (2019). Towards the functional high-resolution coor-dination chemistry of blood plasma human serum albumin. J. Inorg. Biochem..

[24] Zarbock J., Oschkinat H., Hannappel E., Kalbacher H., Voelter W., Holak T.A. (1990). Solution Conformation of Thymosin beta4: A Nuclear Magnetic Resonance and Simulated Annealing Study. Biochemistry.

[25] Emwas A.-H., Szczepski K., Poulson B.G., Chandra K., McKay R.T., Dhahri M., AlAhmari F., Jaremko L., Lachowicz J.I., Jaremko M. (2020). NMR as a “Gold Standard” Method in Drug Design and Discovery. Molecules.

[26] Williamson M.P. (2013). Using chemical shift perturbation to characterise ligand binding. Prog. Nucl. Magn. Reson. Spectrosc..

[27] Low T., Goldstein A.L. (1982). Chemical characterization of thymosin beta 4. J. Biol. Chem..

[28] Mujika J.I., Torre G.D., Formoso E., Grande-Aztatzi R., Grabowski S.J., Exley C., Lopez X. (2018). Aluminum's preferential binding site in proteins: Sidechain of amino acids versus backbone interactions. J. Inorg. Biochem..

[29] Dudev T., Lim C. (2008). Metal Binding Affinity and Selectivity in Metalloproteins: Insights from Computational Studies. Annu. Rev. Biophys..

[30] Ganz T. (2013). Systemic iron homeostasis. Physiol. Rev..

[31] Cazzola M., Della Porta M.G., Malcovati L. (2008). Clinical Relevance of Anemia and Transfusion Iron Overload in Myelodysplastic Syndromes. Hematology.

[32] Shenoy N., Vallumsetla N., Rachmilewitz E., Verma A., Ginzburg Y. (2014). Impact of iron overload and potential benefit from iron chelation in low-risk myelodysplastic syndrome. Blood.

[33] Youssef L.A., Rebbaa A., Pampou S., Weisberg S.P., Stockwell B.R., Hod E.A., Spitalnik S.L. (2018). Increased erythrophago-cytosis induces ferroptosis in red pulp macrophages in a mouse model of transfusion. Blood Am. J. Hematol..

[34] Imoto S., Kono M., Suzuki T., Shibuya Y., Sawamura T., Mizokoshi Y., Sawada H., Ohbuchi A., Saigo K. (2018). Haemin-induced cell death in human monocytic cells is consistent with ferroptosis. Transfus. Apher. Sci..

[35] Soares M.P., Hamza I. (2016). Macrophages and Iron Metabolism. Immunity.

[36] Angeli J.P.F., Schneider M., Proneth B., Tyurina Y.Y., Tyurin V., Hammond V.J., Herbach N., Aichler M., Walch A., Eggenhofer E. (2014). Inactivation of the ferroptosis regulator Gpx4 triggers acute renal failure in mice. Nat. Cell Biol..

[37] Grohm J., Plesnila N., Culmsee C. (2010). Bid mediates fission, membrane permeabilization and perinuclear accumulation of mitochondria as a prerequisite for oxidative neuronal cell death. Brain Behav. Immun..

[38] Jelinek A., Heyder L., Daude M., Plessner M., Krippner S., Grosse R., Diederich W.E., Culmsee C. (2018). Mitochondrial rescue prevents glutathione peroxidase-dependent ferroptosis. Free Radic. Biol. Med..

[39] Neitemeier S., Jelinek A., Laino V., Hoffmann L., Eisenbach I., Eying R., Ganjam G.K., Dolga A., Oppermann S., Culmsee C. (2017). BID links ferroptosis to mitochondrial cell death pathways. Redox Biol..

[40] Wang H., Liu C., Zhao Y., Gao G. (2019). Mitochondria regulation in ferroptosis. Eur. J. Cell Biol..

[41] Gerdes H.-H., Carvalho R.N. (2008). Intercellular transfer mediated by tunneling nanotubes. Curr. Opin. Cell Biol..

[42] Davis D.M., Sowinski S. (2008). Membrane nanotubes: Dynamic long-distance connections between animal cells. Nat. Rev. Mol. Cell Biol..

[43] Zhu D., Tan K.S., Zhang X., Sun A.Y., Sun G.Y., Lee J.C.-M. (2005). Hydrogen peroxide alters membrane and cytoskeleton properties and increases intercellular connections in astrocytes. J. Cell Sci..

[44] Luchetti F., Canonico B., Arcangeletti M., Guescini M., Cesarini E., Stocchi V., Degli Esposti M., Papa S. (2012). Fas Signalling Promotes Intercellular Communication in T Cells. PLoS ONE.

[45] Miotto G., Rossetto M., Di Paolo M.L., Orian L., Venerando R., Roveri A., Vučković A.-M., Travain V.B., Zaccarin M., Zennaro L. (2019). Insight into the mechanism of ferroptosis inhibition by ferrostatin-1. Redox Biol..

[46] Kajarabille N., Latunde-Dada G.O. (2019). Programmed Cell-Death by Ferroptosis: Antioxidants as Mitigators. Int. J. Mol. Sci..

[47] Stockwell B.R., Angeli J.P.F., Bayir H., Bush A., Conrad M., Dixon S.J., Fulda S., Gascón S., Hatzios S.K., Kagan V.E. (2017). Ferroptosis: A Regulated Cell Death Nexus Linking Metabolism, Redox Biology, and Disease. Cell.

[48] Hou Q., Hsu Y.-T. (2005). Bax translocates from cytosol to mitochondria in cardiac cells during apoptosis: Development of a GFP-Bax-stable H9c2 cell line for apoptosis analysis. Am. J. Physiol. Circ. Physiol..

[49] Jungas T., Motta I., Duffieux F., Fanen P., Stoven V., Ojcius D.M. (2002). Glutathione Levels and BAX Activation during Apoptosis Due to Oxidative Stress in Cells Expressing Wild-type and Mutant Cystic Fibrosis Transmembrane Conductance Regulator. J. Biol. Chem..

[50] Chen J., Zhu R.L., Nakayama M., Kawaguchi K., Jin K., Stetler R.A., Simon R.P., Graham S.H. (2002). Expression of the Apoptosis-Effector Gene, Bax, Is Up-Regulated in Vulnerable Hippocampal CA1 Neurons Following Global Ischemia. J. Neurochem..

[51] Krajewski S., Mai J.K., Krajewska M., Sikorska M., Mossakowski M.J., Reed J.C. (1995). Upregulation of bax protein levels in neurons following cerebral ischemia. J. Neurosci..

[52] Wei C., Kumar S., Kim I.-K., Gupta S. (2012). Thymosin beta 4 protects cardiomyocytes from oxidative stress by targeting ant-ioxidative enzymes and anti-apoptotic genes. PLoS ONE.

[53] Kumar S., Gupta S. (2011). Thymosin beta 4 prevents oxidative stress by targeting antioxidant and anti-apoptotic genes in cardiac fibroblasts. PLoS ONE.

[54] Guo S., Wharton W., Moseley P., Shi H. (2007). Heat shock protein 70 regulates cellular redox status by modulating glutathi-one-related enzyme activities. Cell Stress Chaperones.

[55] Papadopoulos M.C., Sun X.Y., Cao J., Mivechi N.F., Giffard R.G. (1996). Over-expression of HSP-70 protects astrocytes from combined oxygen-glucose deprivation. NeuroReport.

[56] Plumier J.-C.L., Krueger A.M., Currie R.W., Kontoyiannis D., Kollias G., Pagoulatos G.N. (1997). Transgenic mice expressing the human inducible Hsp70 have hippocampal neurons resistant to ischemic injury. Cell Stress Chaperon.

[57] Aufricht C., Lu E., Thulin G., Kashgarian M., Siegel N.J., Van Why S.K. (1998). ATP releases HSP-72 from protein aggregates after renal ischemia. Am. J. Physiol. Content.

[58] Bidmon B., Endemann M., Müller T., Arbeiter K., Herkner K., Aufricht C. (2000). Heat shock protein-70 repairs proximal tubule structure after renal ischemia. Kidney Int..

[59] Calabrese V., Copani A., Testa D., Ravagna A., Spadaro F., Tendi E., Nicoletti V., Stella A.G. (2000). Nitric oxide synthase induction in astroglial cell cultures: Effect on heat shock protein 70 synthesis and oxidant/antioxidant balance. J. Neurosci. Res..

[60] McLaughlin B., Hartnett K.A., Erhardt J.A., Legos J.J., White R.F., Barone F.C., Aizenman E. (2003). Caspase 3 activation is essential for neuroprotection in preconditioning. Proc. Natl. Acad. Sci. USA.

[61] Yan L.-J., Christians E.S., Liu L., Xiao X., Sohal R.S., Benjamin I.J. (2002). Mouse heat shock transcription factor 1 deficiency alters cardiac redox homeostasis and increases mitochondrial oxidative damage. EMBO J..

[62] Chiang S.-K., Chen S.-E., Chang L.-C. (2018). A dual role of heme oxygenase-1 in cancer cells. Int. J. Mol. Sci..

[63] Jozkowicz A., Was H., Dulak J. (2007). Heme oxygenase-1 in tumors: Is it a false friend?. Antioxid. Redox Signal..

[64] Hsu Y.-Y., Chen C.-S., Wu S.-N., Jong Y.-J., Lo Y.-C. (2012). Berberine activates Nrf2 nuclear translocation and protects against oxidative damage via a phosphatidylinositol 3-kinase/Akt-dependent mechanism in NSC34 motor neuron-like cells. Eur. J. Pharm. Sci..

[65] Kwon M.-Y., Park E., Lee S.-J., Chung S.W. (2015). Heme oxygenase-1 accelerates erastin-induced ferroptotic cell death. Oncotarget.

[66] Sun X., Ou Z., Chen R., Niu X., Chen D., Kang R., Tang D. (2015). Activation of the p62-Keap1-NRF2 pathway protects against ferroptosis in hepatocellular carcinoma cells. Hepatology.

[67] Gasdaska P.Y., Gasdaska J.R., Cochran S., Powis G. (1995). Cloning and sequencing of a human thioredoxin reductase. FEBS Lett..

[68] Cai L.L., Ruberto R.A., Ryan M.J., Eaton J.K., Schreiber S.L., Viswanathan V.S. (2020). Modulation of ferroptosis sensitivity by TXNRD1 in pancreatic cancer cells. bioRxiv.

[69] Yang L., Wang H., Yang X., Wu Q., An P., Jin X., Liu W., Huang X., Li Y., Yan S. (2020). Auranofin mitigates systemic iron overload and induces ferroptosis via distinct mechanisms. Signal Transduct. Target. Ther..

[70] Low T.L., Mercer R.C. (1984). Isolation and structural studies of porcine, ovine and murine thymosin β4 by high-performance liquid chromatography. J. Chromatogr. A.

[71] Snyder W., Cook M., Nasset E., Karhausen L., Howells G.P., Tipton I. (1975). Report of the task group on reference man.

[72] Torti S.V., Torti F.M. (2013). Iron and cancer: More ore to be mined. Nat. Cancer.

[73] Cheah J.H., Kim S.F., Hester L.D., Clancy K.W., Patterson S.E., Papadopoulos V., Snyder S.H. (2006). NMDA receptor-nitric oxide transmission mediates neuronal iron homeostasis via the GTPase Dexras1. Neuron.

[74] Choi D.W. (1988). Glutamate neurotoxicity and diseases of the nervous system. Neuron.

[75] Murphy T.H., Miyamoto M., Sastre A., Schnaar R.L., Coyle J.T. (1989). Glutamate toxicity in a neuronal cell line involves inhi-bition of cystine transport leading to oxidative stress. Neuron.

[76] Forciniti S., Greco L., Grizzi F., Malesci A., Laghi L. (2020). Iron metabolism in cancer progression. Int. J. Mol. Sci..

[77] Hametner S., Wimmer I., Haider L., Pfeifenbring S., Brück W., Lassmann H. (2013). Iron and neurodegeneration in the multiple sclerosis brain. Ann. Neurol..

[78] Muhoberac B.B., Vidal R. (2013). Abnormal iron homeostasis and neurodegeneration. Front. Aging Neurosci..

[79] Yang W.S., Stockwell B.R. (2015). Ferroptosis: Death by Lipid Peroxidation. Trends Cell Biol..

[80] Tan S., Schubert D., Maher P. (2001). Oxytosis: A Novel Form of Programmed Cell Death. Curr. Top. Med. Chem..

[81] Kang Y., Tiziani S., Park G., Kaul M., Paternostro G. (2014). Cellular protection using Flt3 and PI3Kα inhibitors demonstrates multiple mechanisms of oxidative glutamate toxicity. Nat. Commun..

[82] Morrison B., Pringle A., McManus T., Ellard J., Bradley M., Signorelli F., Iannotti F., E Sundstrom L. (2002). L -Arginyl-3,4-Spermidine is neuroprotective in several in vitro models of neurodegeneration and in vivo ischaemia without suppressing synaptic transmission. Br. J. Pharmacol..

[83] Sundstrom L., Pringle A., Morrison B., Bradley M. (2005). Organotypic cultures as tools for functional screening in the CNS. Drug Discov. Today.

[84] Zhu Z., Zhang Y., Huang X., Can L., Zhao X., Wang Y., Xue J., Cheng M., Zhu L. (2021). Thymosin beta 4 alleviates non-alcoholic fatty liver by inhibiting ferroptosis via up-regulation of GPX4. Eur. J. Pharmacol..

[85] Alghrably M., Dudek D., Emwas A.-H., Jaremko M., Rowińska-Żyrek M. (2020). Copper(II) and Amylin Analogues: A Complicated Relationship. Inorg. Chem..

[86] Sharfalddin A.A., Emwas A.H., Jaremko M., Hussien M.A. (2021). Transition metal complexes of 6-mercaptopurine: Characterization, Theoretical calculation, DNA-Binding, molecular docking, and anticancer activity. Appl. Organomet. Chem..

[87] Glasoe P.K., Long F.A. (1960). USE OF GLASS ELECTRODES TO MEASURE ACIDITIES IN DEUTERIUM OXIDE1,2. J. Phys. Chem..

[88] Willker W., Wollborn U., Leibfritz D. (1993). Exact Measurement of 3JCH Coupling Constants Using Proton-Detected Editing and Selection Sequences. J. Magn. Reson. Ser. B.

[89] Jorgensen W.L., Maxwell D.S., Tirado-Rives J. (1996). Development and Testing of the OPLS All-Atom Force Field on Conformational Energetics and Properties of Organic Liquids. J. Am. Chem. Soc..

[90] (2020). MacroModel.

[91] Chang G., Guida W.C., Still W.C. (1989). An internal-coordinate Monte Carlo method for searching conformational space. J. Am. Chem. Soc..

[92] Polak E., Ribiere G. (1969). Note sur la convergence de méthodes de directions conjuguées. ESAIM Math. Model. Numer. Anal..

[93] Ponder J.W., Richards F.M. (1987). An efficient newton-like method for molecular mechanics energy minimization of large mole-cules. J. Comput. Chem..

[94] Horn H.W., Swope W.C., Pitera J.W., Madura J., Dick T.J., Hura G.L., Head-Gordon T. (2004). Development of an improved four-site water model for biomolecular simulations: TIP4P-Ew. J. Chem. Phys..

[95] Best R.B., Hummer G. (2009). Optimized Molecular Dynamics Force Fields Applied to the Helix−Coil Transition of Polypeptides. J. Phys. Chem. B.

[96] Lindorff-Larsen K., Piana S., Palmo K., Maragakis P., Klepeis J.L., Dror R.O., Shaw D.E. (2010). Improved side-chain torsion potentials for the Amber ff99SB protein force field. Proteins Struct. Funct. Bioinform..

[97] Li P., Song L.F., Merz K.M. (2015). Parameterization of highly charged metal ions using the 12-6-4 LJ-type nonbonded model in explicit water. J. Phys. Chem. B.

[98] Darden T., York D., Pedersen L. (1993). Particle mesh Ewald: An *N*⋅log(*N*) method for Ewald sums in large systems. J. Chem. Phys..

[99] Hess B., Bekker H., Berendsen H.J., Fraaije J.G. (1997). LINCS: A linear constraint solver for molecular simulations. J. Comput. Chem..

[100] Feenstra K.A., Hess B., Berendsen H.J. (1999). Improving efficiency of large time-scale molecular dynamics simulations of hydrogen-rich systems. J. Comput. Chem..

[101] Parrinello M., Rahman A. (1981). Polymorphic transitions in single crystals: A new molecular dynamics method. J. Appl. Phys..

[102] Durzyńska J., Barton E. (2014). IGF expression in HPV-related and HPV-unrelated human cancer cells. Oncol. Rep..

[103] Livak K.J., Schmittgen T.D. (2001). Analysis of relative gene expression data using real-time quantitative PCR and the 2^−ΔΔCT^ method. Methods.

