# Supplementary material for: Thymosin β4 Is an Endogenous Iron Chelator and Molecular Switcher of Ferroptosis
Source: Int J Mol Sci. 2022 Jan 4;23(1):551. doi: 10.3390/ijms23010551 (PMC8745404; doi:10.3390/ijms23010551)
Supplement: Supplementary file 1 [file ijms-23-00551-s001.zip › ijms-1529599-supplementary.pdf]

**ELECTRONIC SUPPLEMENATRY MATERIAL**

# Thymosin β4 is an endogenous iron chelator and molecular switcher of ferroptosis

*Joanna I. Lachowicz[1], Giusi Pichiri,[1,*] Marco Piludu[2,*], Sara Fais[3], Germano Orrù[3], Terenzio Congiu[1], Monica Piras[1], Gavino Faa[1], Daniela Fanni[1], Gabriele dalla Torre[4], Xabier Lopez[4], Kousik Chandra, [5] Kacper Szczepski, [5] Lukasz Jaremko, [5] Mitra Ghosh, [5] Abdul-Hamid Emwas,[6] Castagnola Massimo[7,8], Mariusz Jaremko[5,*] Ewald Hannappel[9] and Pierpaolo Coni[1].*

[1]*Department of Medical Sciences and Public Health, University of Cagliari, Cittadella Universitaria, 09042 Monserrato, Italy*

[2]*Department of Biomedical Sciences, University of Cagliari, Cittadella Universitaria, 09042 Monserrato, Italy*

[3]*Department of Surgical Science, OBL Oral Biotechnology Laboratory, University of Cagliari, Cagliari Italy*

[4]*Kimika Fakultatea, Euskal Herriko Unibertsitatea UPV/EHU, Donostia International Physics Center (DIPC), P.K. 1072 Donostia Euskadi, San Sebastian, 20080, Spain*

[5]*Division of Biological and Environmental Sciences and Engineering (BESE), King Abdullah University of Science and Technology (KAUST), 23955-6900 Thuwal, Saudi Arabia.*

[6]*Core Labs, King Abdullah University of Science and Technology (KAUST), 23955-6900 Thuwal, Saudi Arabia.*

[7]*Institute of Chemistry of Molecular Recognition, National Research Council (Consiglio Nazionale delle Ricerche), Rome, Italy*

[8]*Laboratory of Proteomics and Metabolomics, IRCCS, Santa Lucia Foundation, Rome, Italy*

[9]*Friedrich-Alexander-University Erlangen-Nuremberg, Institute of Biochemistry, Erlangen, Germany*

**Corresponding Authors: Marco Piludu (piludu@unica.it); Mariusz Jaremko (Mariusz.jaremko@kaust.edu.sa); Giuseppina Pichiri (pichiri@unica.it);*

**TABLE OF CONTEST**

**Figure S1.** The assigned $^{13}C$-$^{1}H$ HSQC of **free thymosin (Tβ4)** in $D_2O$. 2048 and 512 data points were collected in proton and carbon dimensions, respectively, which correspond to 125 ms and 18 ms acquisition time in the respective dimension. The data were apodized with sine function and zero filled with 4096 and 1024 points in proton and carbon dimensions, respectively. The recycle delay was kept for 1 second. The total measurement time was 300 minutes.

**Figure S2.** The overlay of the two-dimensional $^{13}C$-$^{1}H$ HSQC spectra in **free Tβ4 form (red)** and with **$Fe^{2+}$/Thymosin** molar ratio 0.2:1 **(yellow)**. Peptide concentration 1.48 mM. The upfield shifted carbon region are shown here. The highlighted residues in the blue dotted circles shows intensity change. No change in chemical shifts were observed.

**Figure S3.** The overlay of the two-dimensional $^{13}C$-$^{1}H$ HSQC spectra in **free Tβ4 form (red)** and with **$Fe^{2+}$/Thymosin** molar ratio 0.2:1 **(yellow)**. The downfield shifted carbon region is shown here. The highlighted residues in the blue dotted circles show intensity change. No change in chemical shifts were observed.

**Figure S4.** The percentage change in intensities from apo to molar ratio 0.2:1 Fe(II)/Thymosine are plotted for the distinct residues from the two-dimensional $^{13}C$-$^{1}H$ HSQC spectra. The red line represents the mean + standard deviation value. Beyond this, the existing residues are considered as major changes.

**Figure S5.** The overlay of the two-dimensional $^{13}C$-$^{1}H$ HSQC spectra in **free Tβ4 form (red)** and with **$Fe^{3+}$/Thymosin** molar ratio 0.2:1 **(yellow)**. The upfield shifted carbon region are shown here. The highlighted residues in the blue dotted circles show intensity changes. No changes in chemical shifts are observed.

**Figure S6.** The overlay of the two-dimensional $^{13}C$-$^{1}H$ HSQC spectra in **free Tβ4 form (red)** and with **$Fe^{3+}$/Thymosin** molar ratio 0.2:1 **(yellow)**. The downfield shifted carbon region are shown here. The highlighted residues in the blue dotted circles show intensity change. No change in chemical shifts were observed.

**Figure S7.** The percentage change in intensities from apo to molar ratio 0.2:1 Fe(III):Thymosine are plotted for the distinct residues from the two-dimensional $^{13}C$-$^{1}H$ HSQC spectra. The red line represents the mean + standard deviation value. Beyond this, the existing residues are considered as a major change.

**Figure S8.** EPR spectra of free Tb4 (0.3 mM) and A) iron (II) and B) iron (III) in water; pH=7.4 (fixed by the addition of NaOH); metal to peptide molar ratio 1:1.

**Figure S9.** CD spectra of free Tβ4 and its iron (II) and iron (III) adducts in Tris buffer (pH 7.5).

**Figure S10.** The assigned $^{13}C$-$^{1}H$ HSQC of free thymosin Tβ4 in $D_2O$. 2048 and 360 data points were collected in proton and carbon dimensions respectively, which correspond to 125 ms and 12.7ms acquisition time in the respective dimension. The data were apodized with sine function and zero filled with 4096 and 1024 points in proton and carbon dimensions, respectively. The recycle delay was kept for 1 second. The total measurement time was 110 minutes.

**Figure S11.** The assigned $^{13}C$-$^{1}H$ TOCSY-HSQC of free thymosin Tβ4 in $D_2O$. Mixing time was kept at 16ms. 2048 and 360 data points were collected in proton and carbon dimensions, respectively, which correspond to 125 ms and 16.2ms acquisition time in the respective dimension. The data were apodized with sine function and zero filled with 4096 and 1024 points in proton and carbon dimensions, respectively. The recycle delay was kept for 1.5 seconds. The total measurement time was 240 minutes.

**Figure S12.** The overlay of the one dimension proton spectra with different Thymosin:Al ratios.

**Figure S13.** The intensities are plotted for the 48 distinct residues from the two-dimensional $^{13}$C-$^{1}$H HSQC spectra as a function of the Thymosin:Al ratio.

**Figure S14.** The chemical shift perturbation (CSP) plot for the distinct $C^{\alpha}$-$H^{\alpha}$ residues from the overlay of the two-dimensional $^{13}$C-$^{1}$H HSQC spectra in free form and with Thymosin:Al ratio 1:5.

**Figure S15.** The chemical shift perturbation (CSP) plot for the distinct C□-H□ and other side chainsfrom the overlay of the two-dimensional $^{13}$C-$^{1}$H HSQC spectra in free form and with Thymosin:Al ratio 1:5.

**Table S1.** Average structural parameters for the three MD simulations.

**Table S2.** Occupancies and average number of residues involved in eight different secondary structure motifs.

**Table S3.** Multistep equilibration scheme with decreasing force constants employed in the molecular dynamics study. FC: Force constants. The subscript bb refers to backbone force constants.

**Figure S16.** Secondary structure analysis according to the DSSP definitions of Tβ4$^{N-C}$.

**Figure S17.** Secondary structure analysis according to the DSSP definitions of Tβ4$^{N-mid}$.

**Figure S18.** Secondary structure analysis according to the DSSP definitions of Tβ4$^{N-N}$.

**Figure S19.** Electron micrographs of a mitotic J774 cell. Arrows indicate the condensed packed chromatin of the chromosomes.

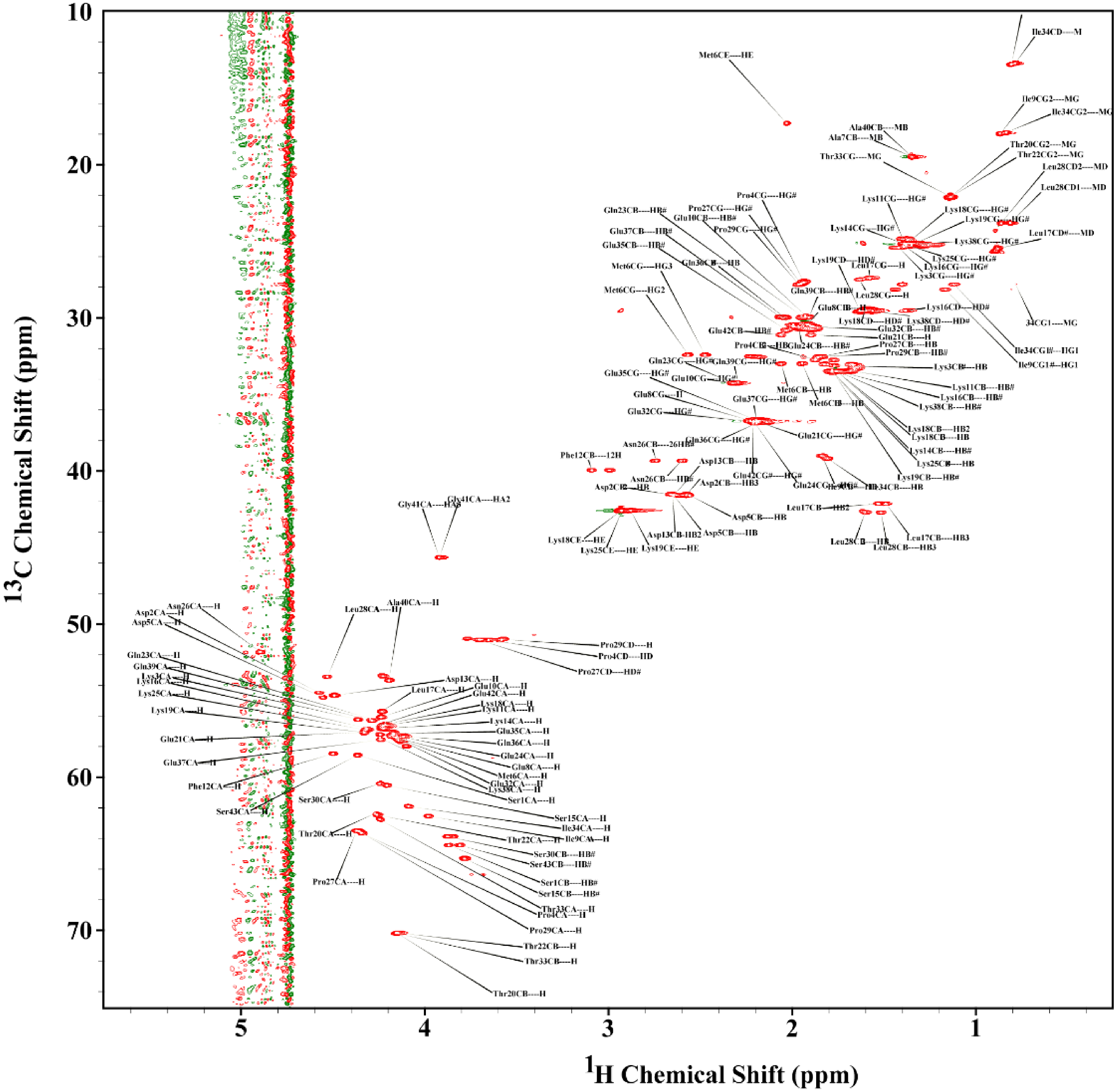


**Figure S1.** The assigned $^{13}$C-$^{1}$H HSQC of **free thymosin (Tβ4)** in $D_2O$. 2048 and 512 data points were collected in proton and carbon dimensions, respectively, which correspond to 125 ms and 18 ms acquisition time in the respective dimension. The data were apodized with sine function and zero filled with 4096 and 1024 points in proton and carbon dimensions, respectively. The recycle delay was kept for 1 second. The total measurement time

was 300 minutes.

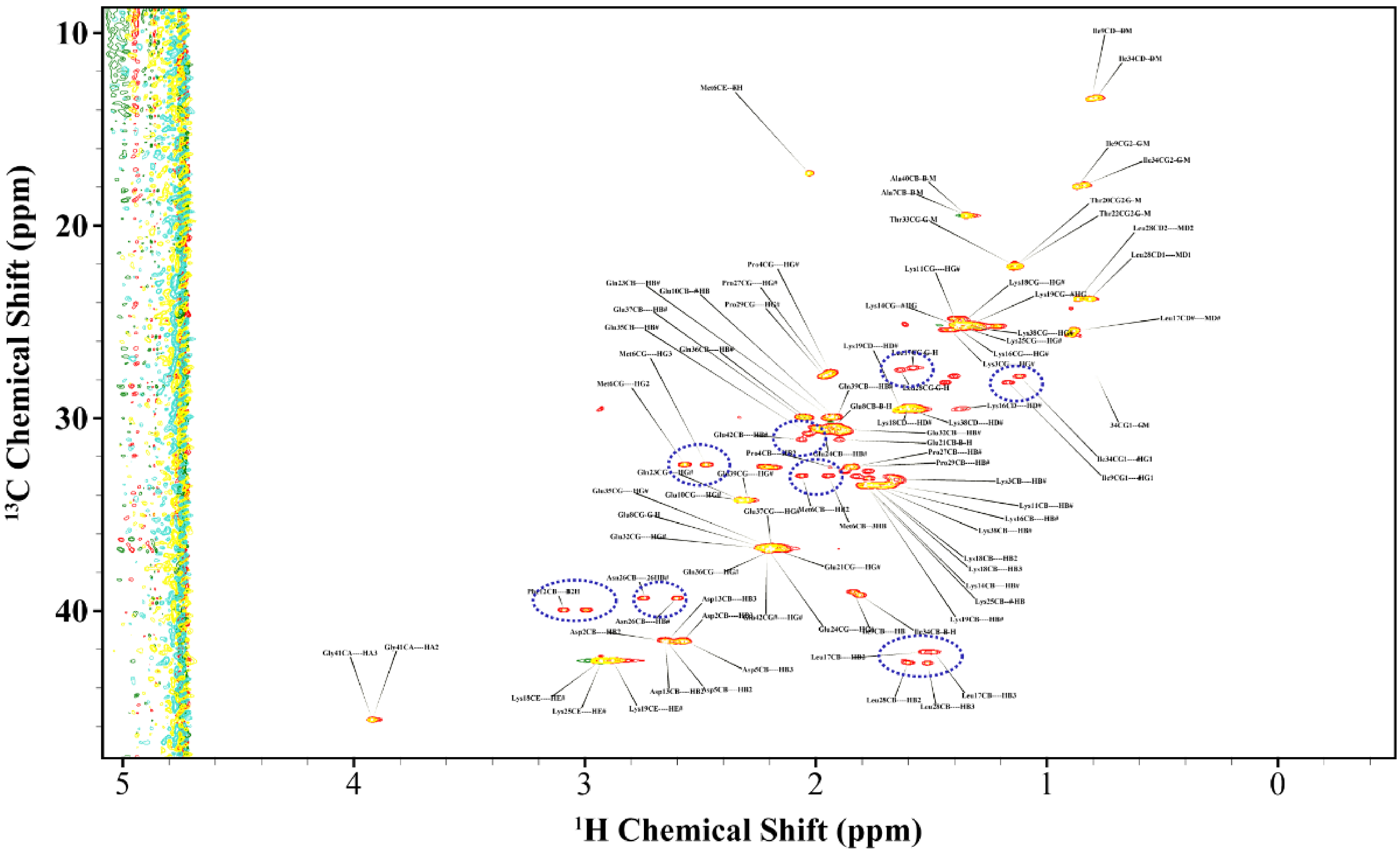


**Figure S2.** The overlay of the two-dimensional $^{13}C$-$^{1}H$ HSQC spectra in **free Tβ4 form (red)** and with **$Fe^{2+}$/Thymosin** molar ratio 0.2:1 **(yellow)**. Peptide concentration 1.48 mM. The upfield shifted carbon region are shown here. The highlighted residues in the blue dotted circles shows intensity change. No change in chemical shifts were observed.

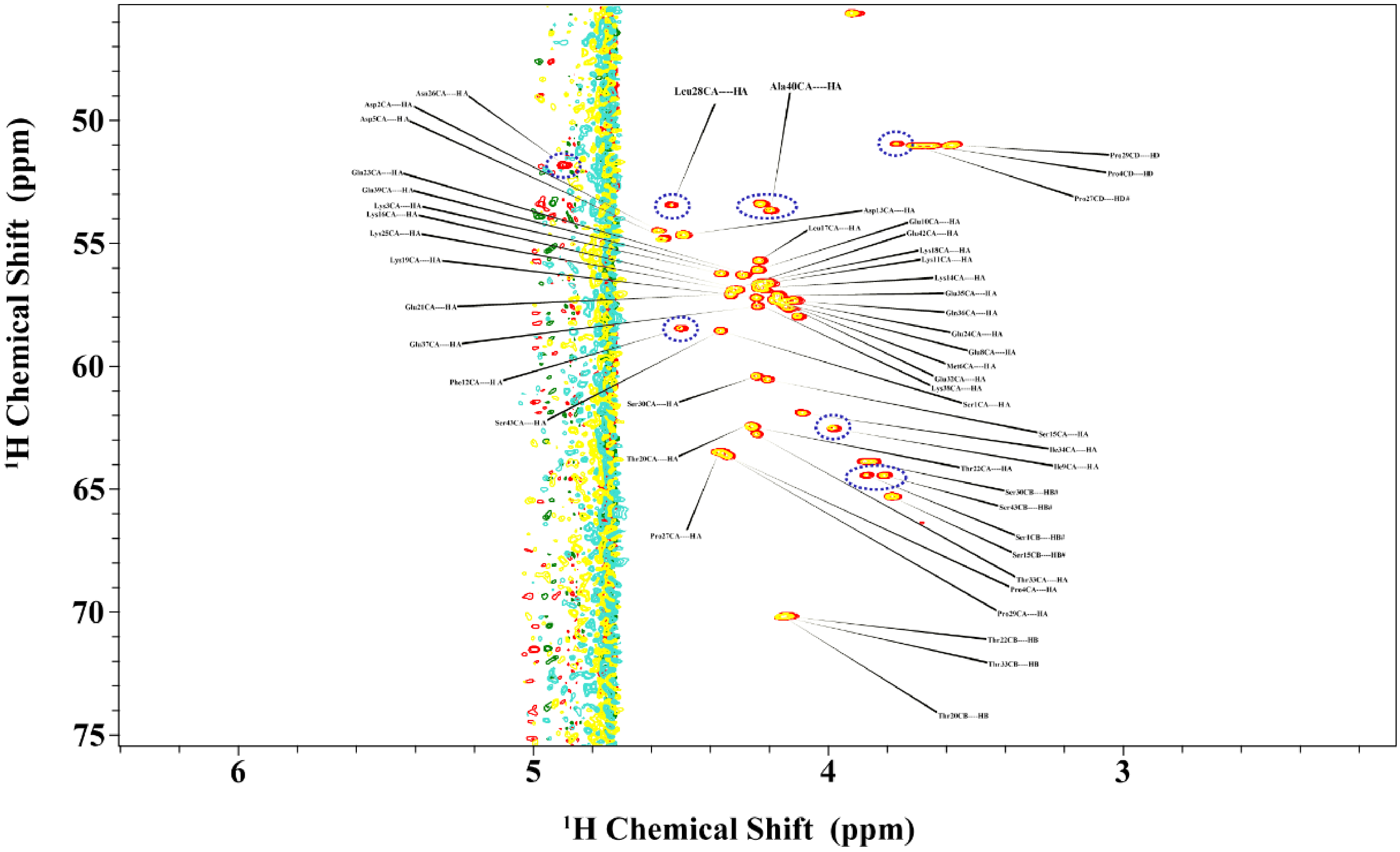


**Figure S3.** The overlay of the two-dimensional $^{13}C$-$^{1}H$ HSQC spectra in **free Tβ4 form (red)** and with **$Fe^{2+}$/Thymosin** molar ratio 0.2:1 **(yellow)**. The downfield shifted carbon region is shown here. The highlighted residues in the blue dotted circles show intensity change. No change in chemical shifts were observed.

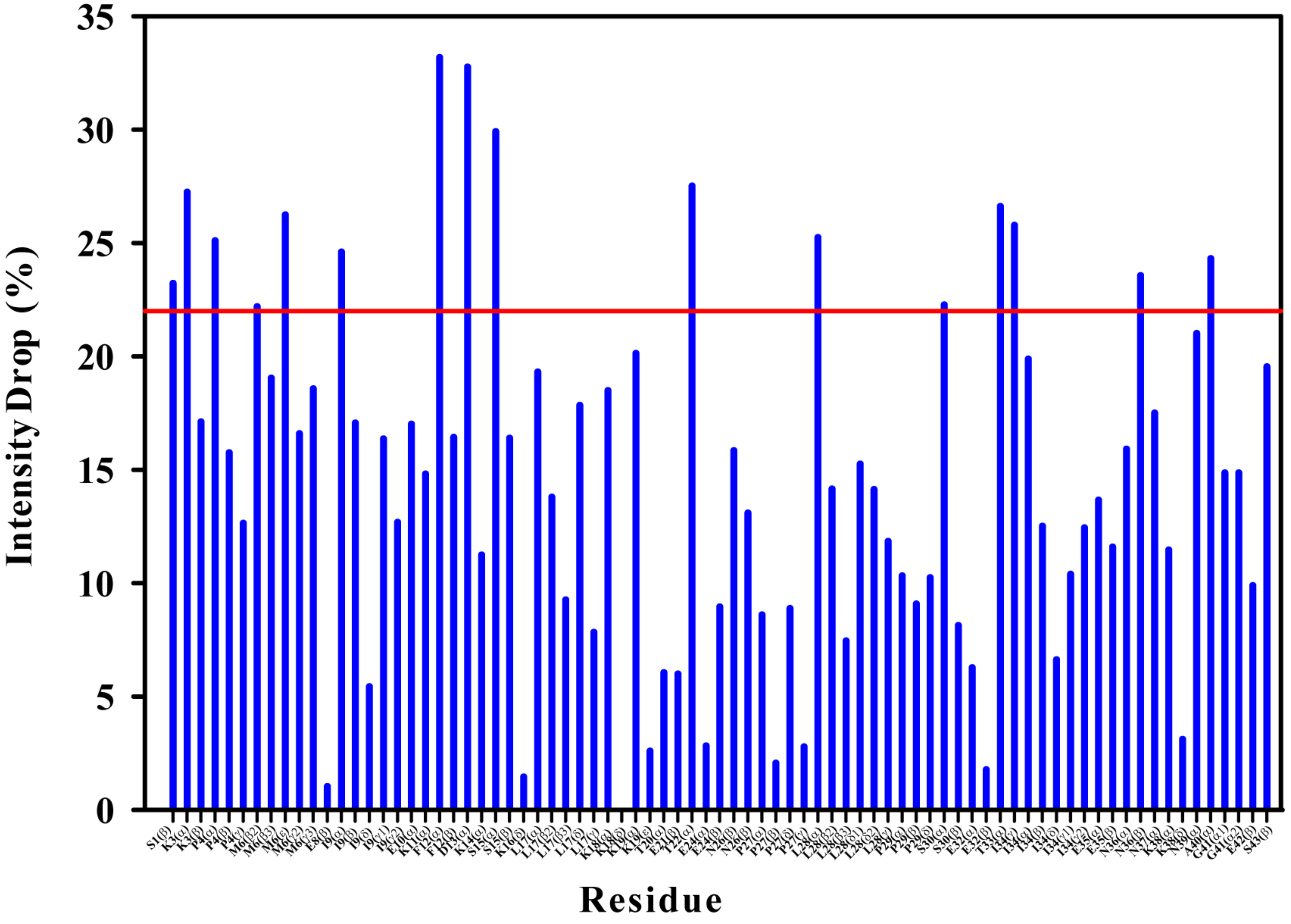


**Figure S4.** The percentage change in intensities from apo to molar ratio 0.2:1 Fe(II)/Thymosine are plotted for the distinct residues from the two-dimensional $^{13}C$-$^{1}H$ HSQC spectra. The red line represents the mean + standard deviation value. Beyond this, the existing residues are considered as major changes.

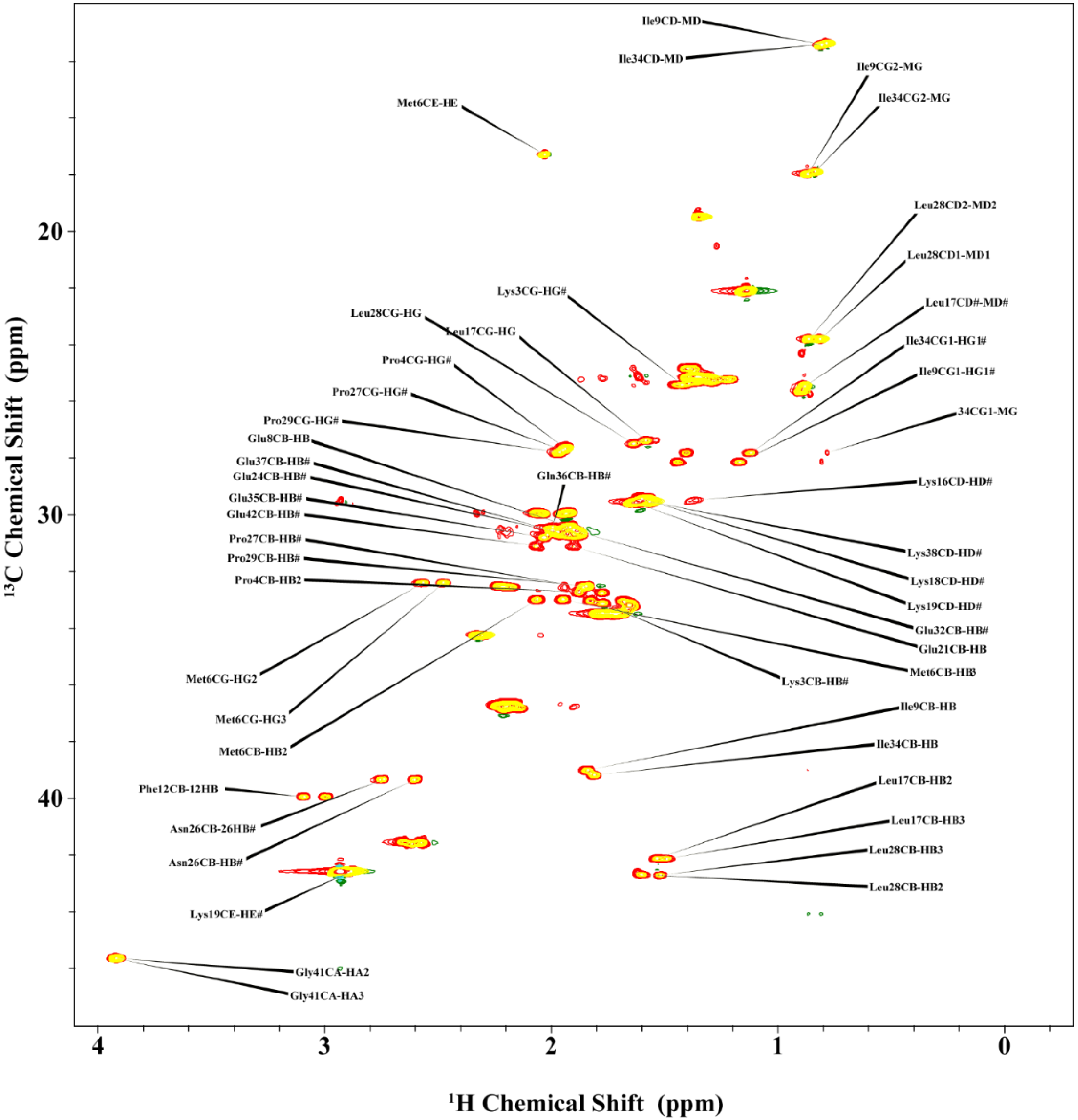


**Figure S5.** The overlay of the two-dimensional $^{13}C$-$^{1}H$ HSQC spectra in **free Tβ4 form (red)** and with **$Fe^{3+}$/Thymosin** molar ratio 0.2:1 **(yellow)**. The upfield shifted carbon region are shown here. The highlighted residues in the blue dotted circles show intensity changes. No changes in chemical shifts are observed.

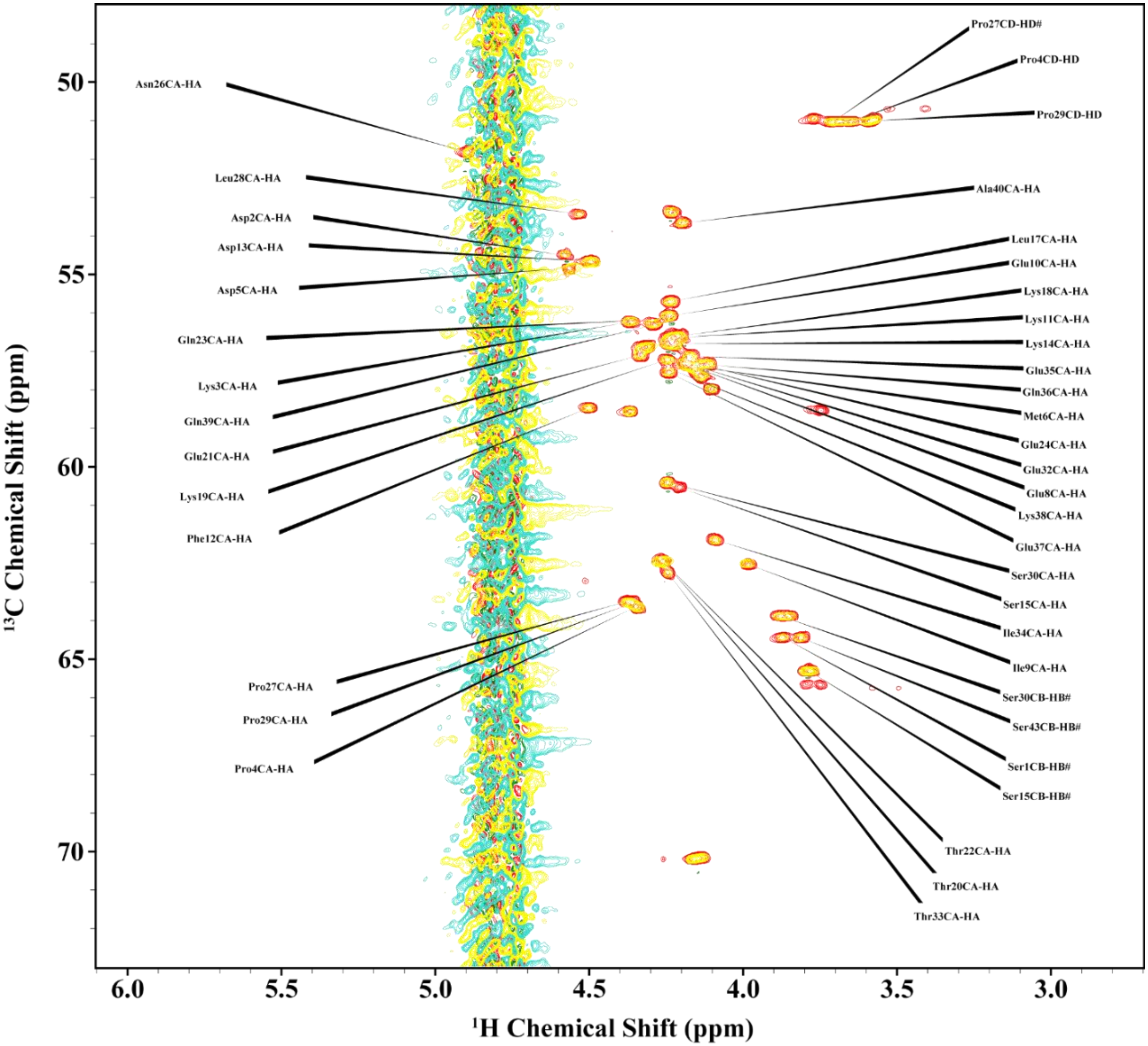


**Figure S6.** The overlay of the two-dimensional $^{13}C$-$^{1}H$ HSQC spectra in **free Tβ4 form (red)** and with **$Fe^{3+}$/Thymosin** molar ratio 0.2:1 **(yellow)**. The downfield shifted carbon region are shown here. The highlighted residues in the blue dotted circles show intensity change. No change in chemical shifts were observed.

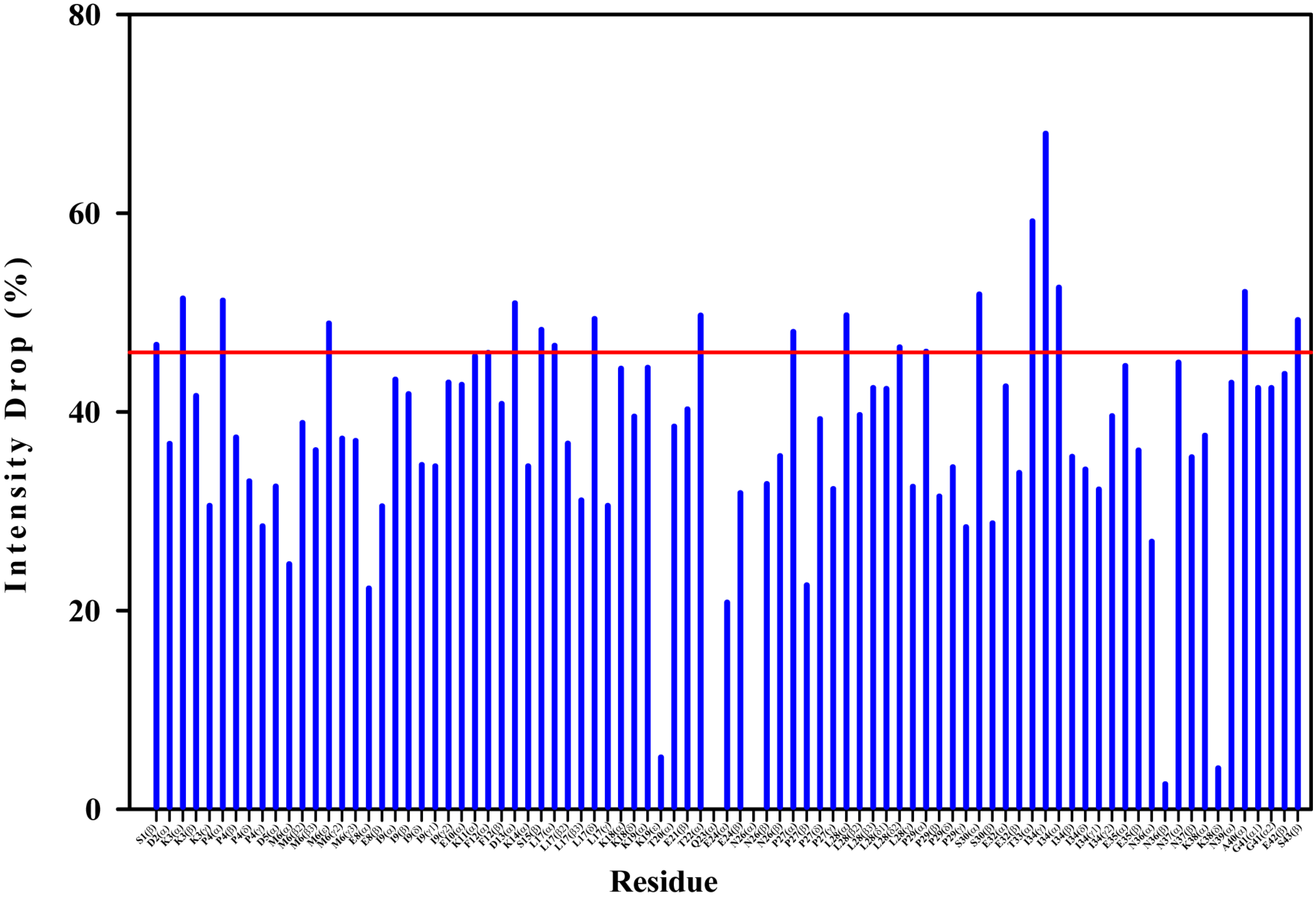


**Figure S7.** The percentage change in intensities from apo to molar ratio 0.2:1 Fe(III):Thymosine are plotted for the distinct residues from the two-dimensional $^{13}C$-$^{1}H$ HSQC spectra. The red line represents the mean + standard deviation value. Beyond this, the existing residues are considered as a major change.

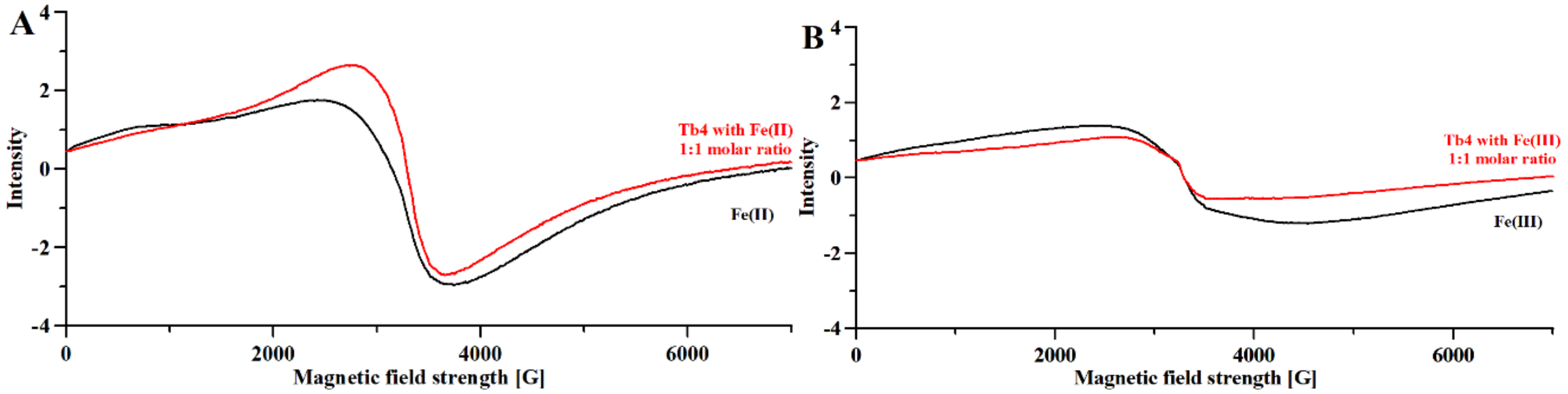


**Figure S8.** EPR spectra of free Tb4 (0.3 mM) and A) iron (II) and B) iron (III) in water; pH=7.4 (fixed by the addition of NaOH); metal to peptide molar ratio 1:1.

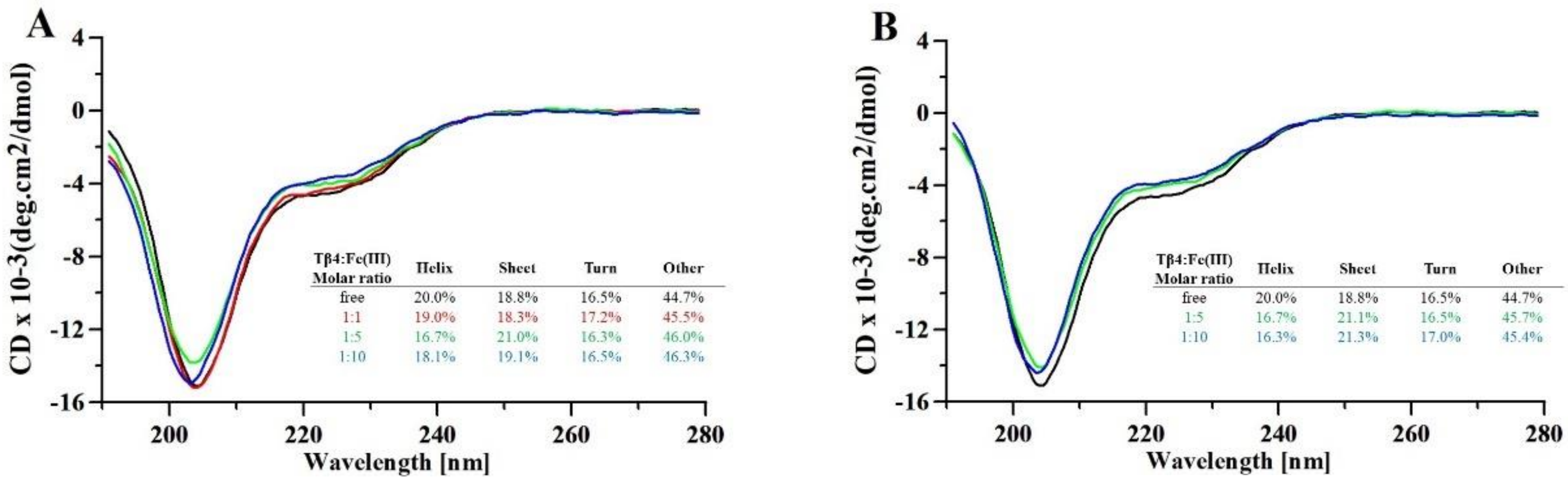


**Figure S9.** CD spectra of free Tβ4 and its iron (II) and iron (III) adducts in Tris buffer (pH 7.5).

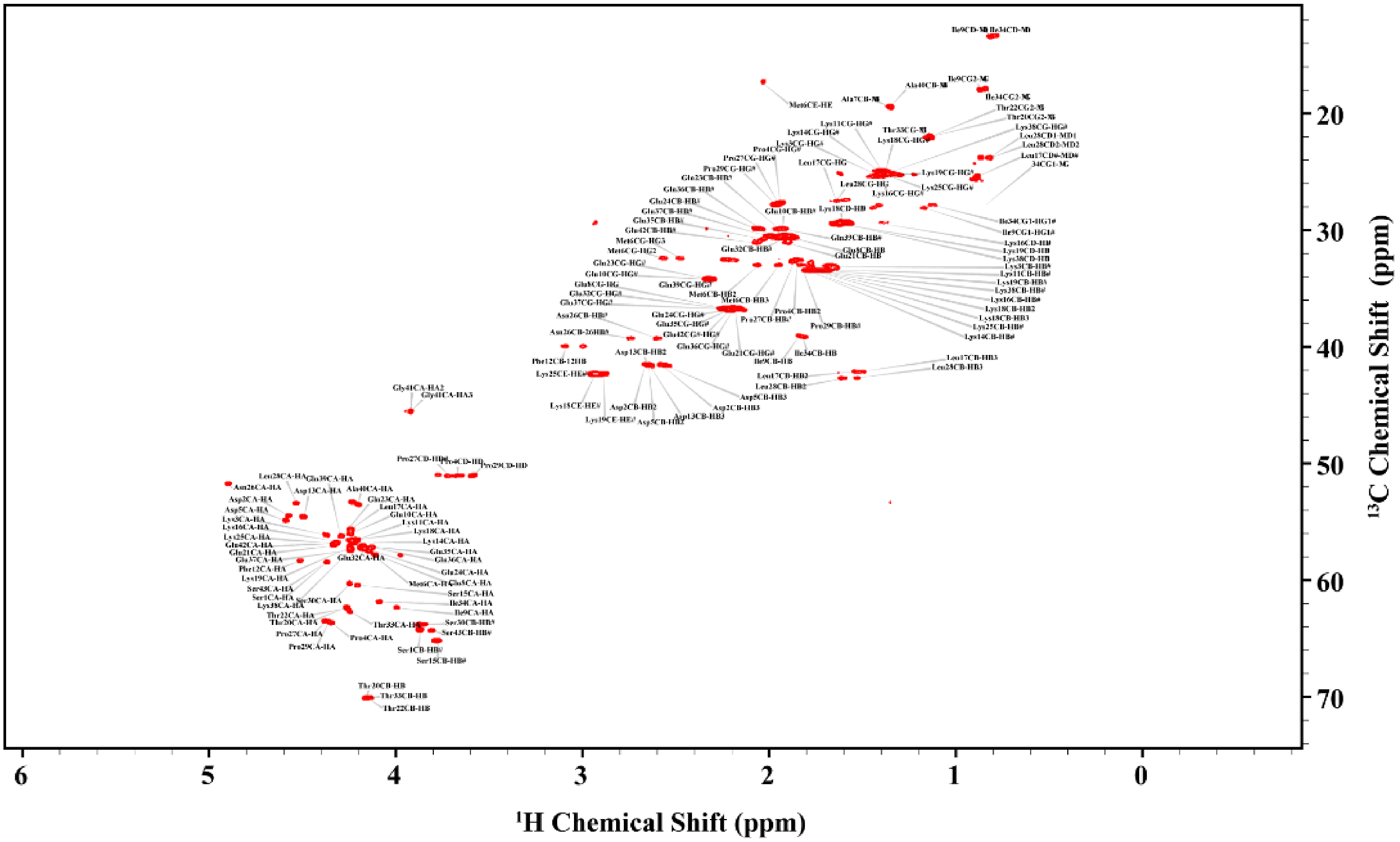


**Figure S10.** The assigned $^{13}C$-$^{1}H$ HSQC of free thymosin Tβ4 in $D_2O$. 2048 and 360 data points were collected in proton and carbon dimensions respectively, which correspond to 125 ms and 12.7ms acquisition time in the respective dimension. The data were apodized with sine function and zero filled with 4096 and 1024 points in proton and carbon dimensions, respectively. The recycle delay was kept for 1 second. The total measurement time was 110 minutes.

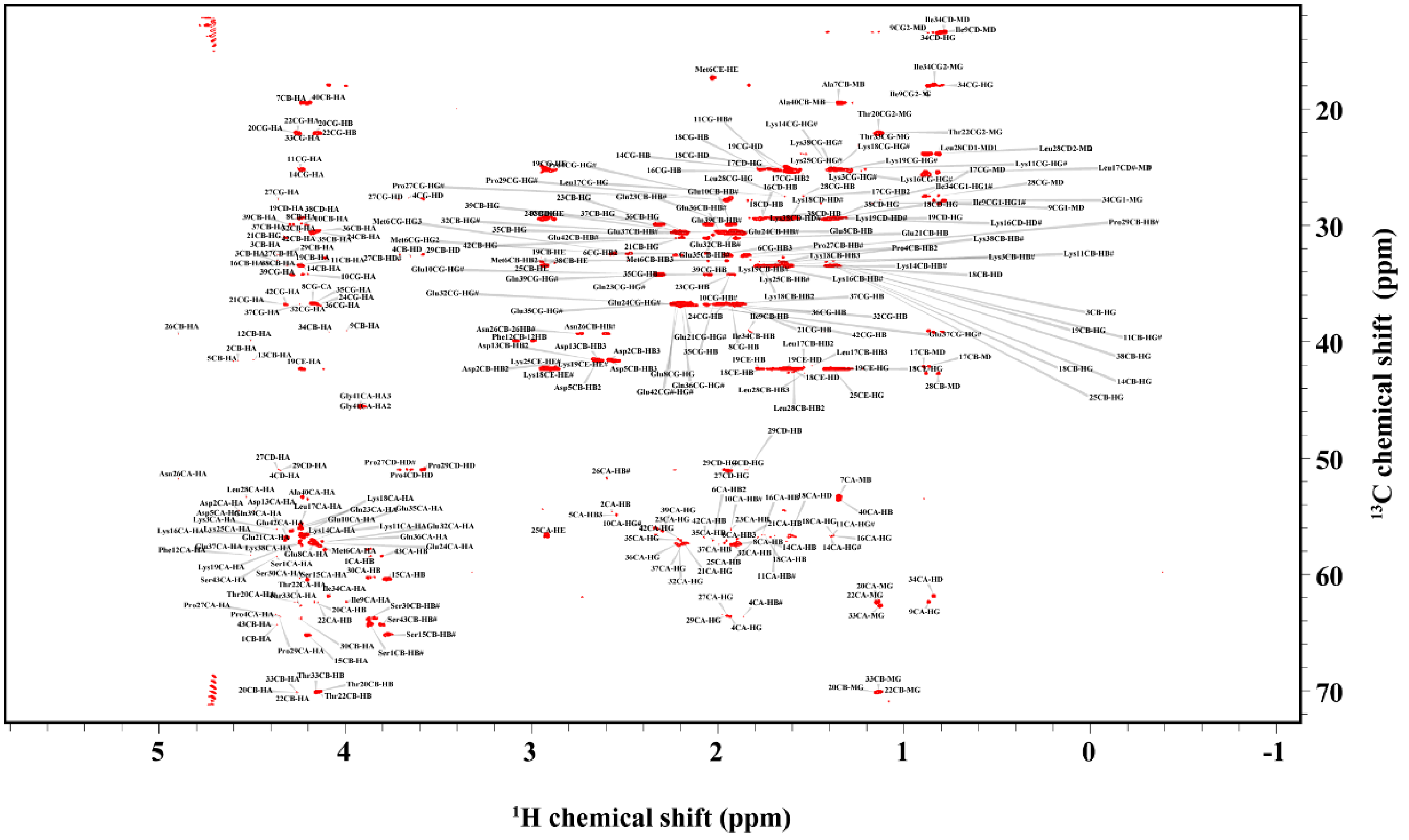


**Figure S11.** The assigned $^{13}$C-$^{1}$H TOCSY-HSQC of free thymosin Tβ4 in $D_2O$. Mixing time was kept at 16ms. 2048 and 360 data points were collected in proton and carbon dimensions, respectively, which correspond to 125 ms and 16.2ms acquisition time in the respective dimension. The data were apodized with sine function and zero filled with 4096 and 1024 points in proton and carbon dimensions, respectively. The recycle delay was kept for 1.5 seconds. The total measurement time was 240 minutes.

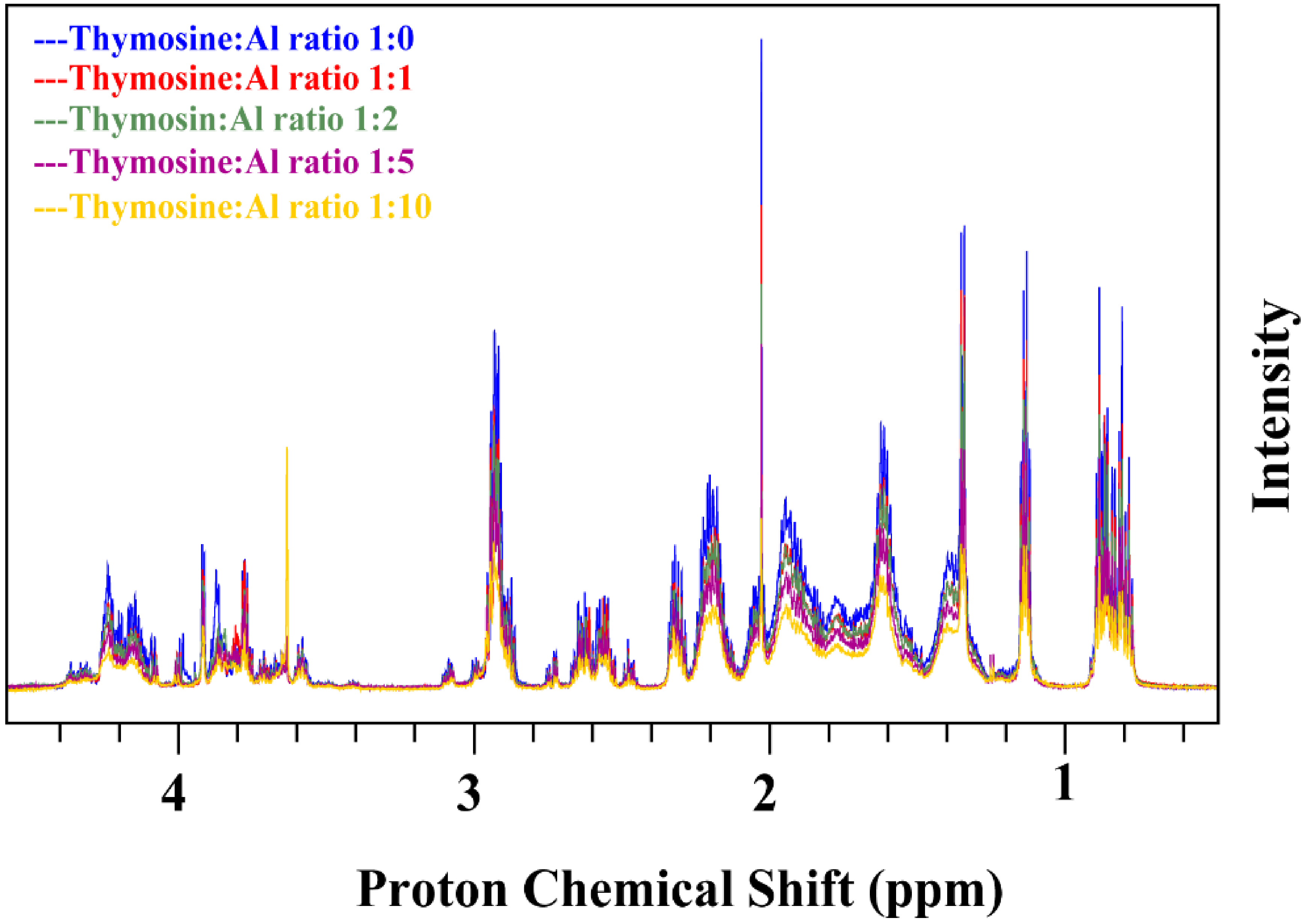


**Figure S12.** The overlay of the one dimension proton spectra with different Thymosin:Al ratios.

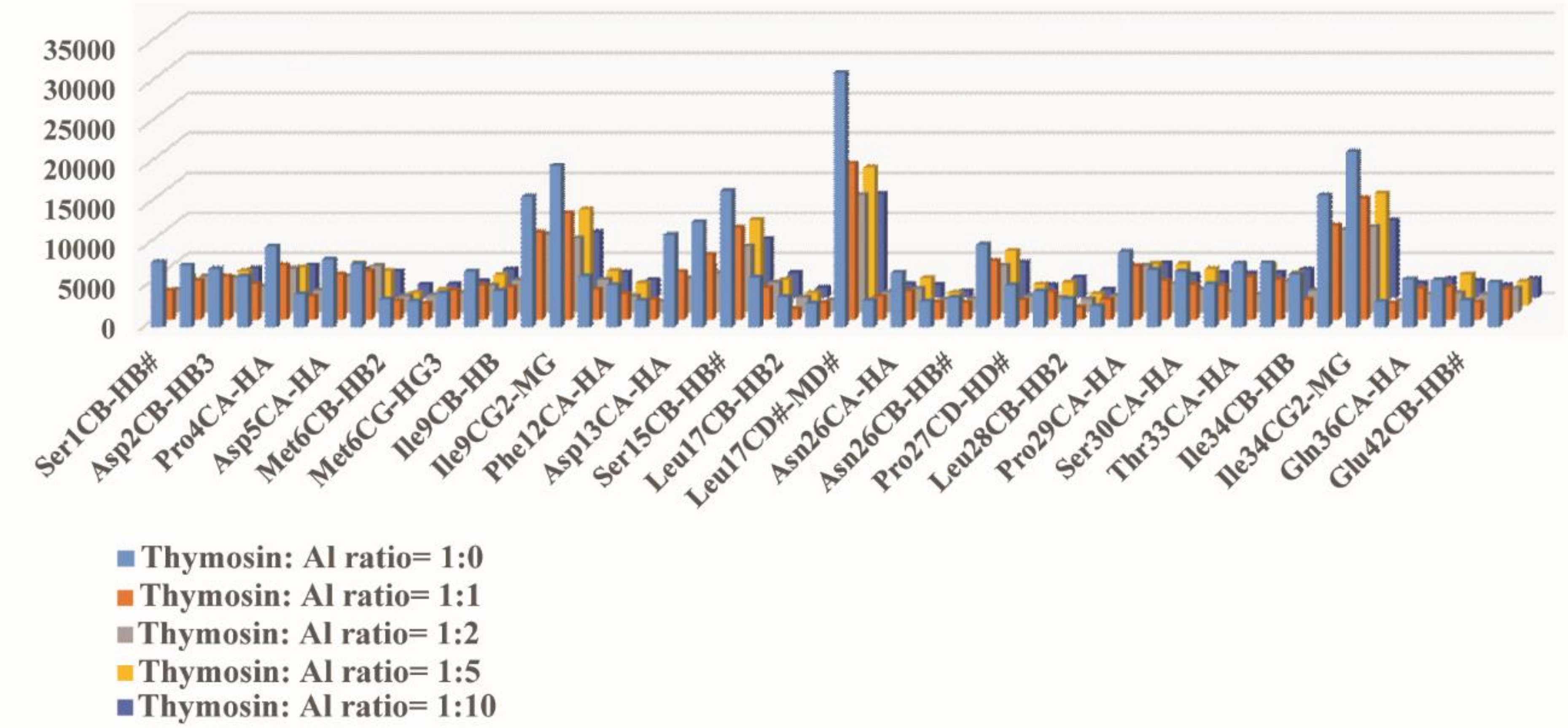


**Figure S13.** The intensities are plotted for the 48 distinct residues from the two-dimensional $^{13}C$-$^{1}H$ HSQC spectra as a function of the Thymosin:Al ratio.

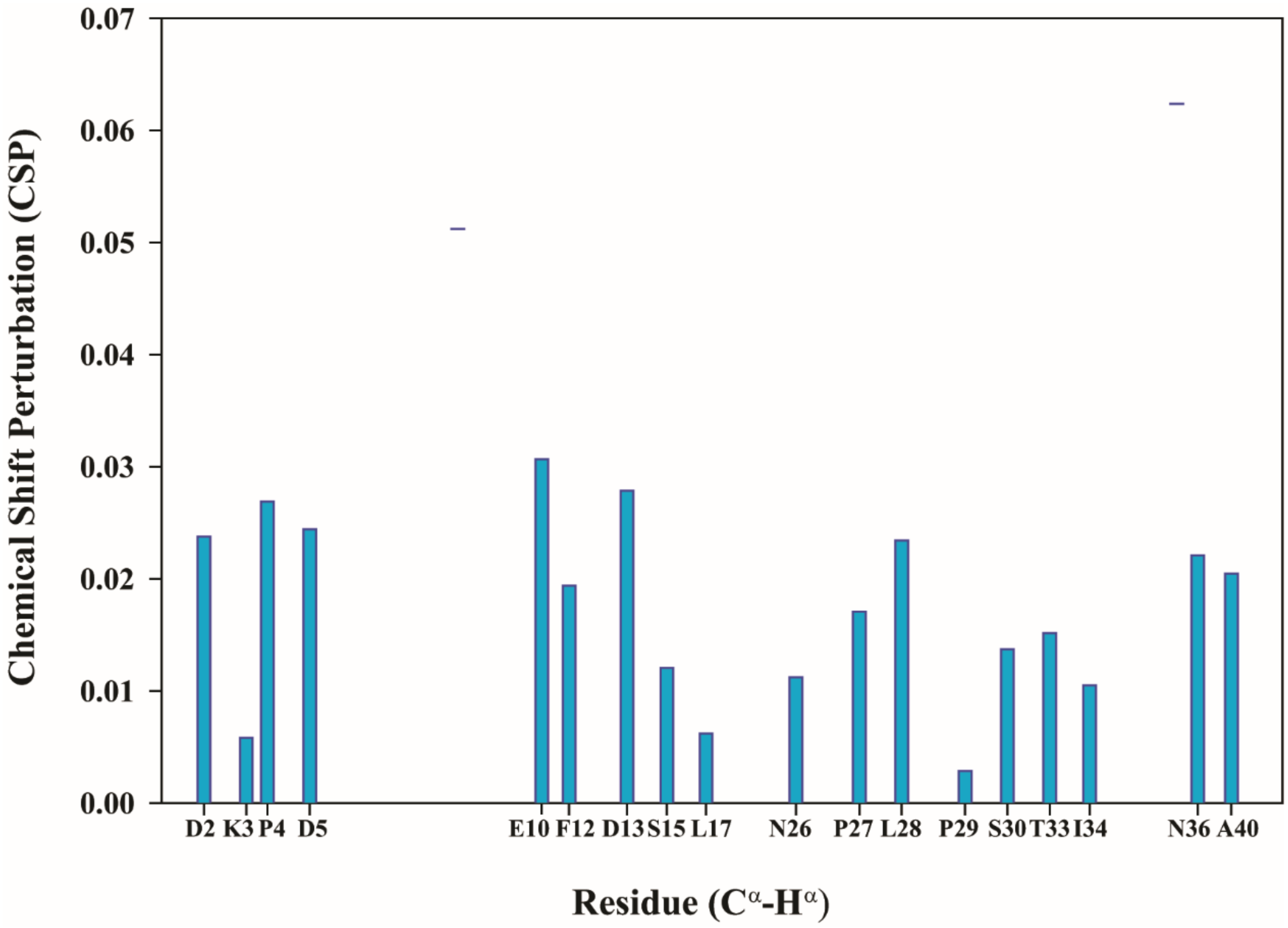


**Figure S14.** The chemical shift perturbation (CSP) plot for the distinct $C^{\alpha}$-$H^{\alpha}$ residues from the overlay of the two-dimensional $^{13}C$-$^{1}H$ HSQC spectra in free form and with Thymosin:Al ratio 1:5.

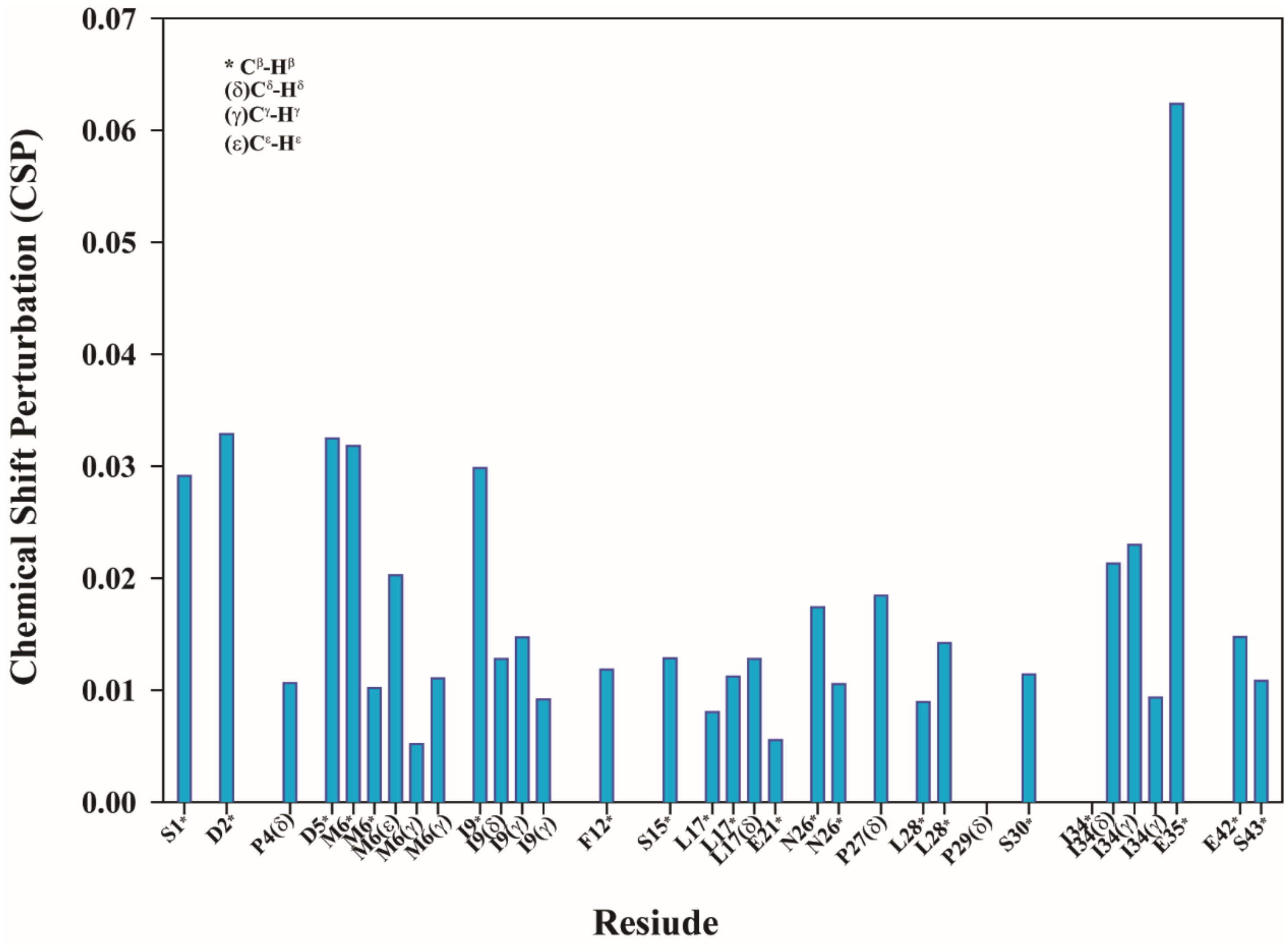


**Figure S15.** The chemical shift perturbation (CSP) plot for the distinct C□-H□ and other side chainsfrom the overlay of the two-dimensional $^{13}C$-$^{1}H$ HSQC spectra in free form and  with Thymosin:Al ratio 1:5.

**Table S1.** Average structural parameters for the three MD simulations.

| Al-thymosin complex | RMSD (Å) | Rg (Å) | End to end distance (Å) | H-bonds |
|---|---|---|---|---|
| **$TB4^{N\text{-}C}$** | 0.92 | 1.15 | 22.27 | 12.06 |
| **$TB4^{N\text{-}mid}$** | 0,89 | 1.11 | 24.37 | 15.28 |
| **$TB4^{N\text{-}N}$** | 1.07 | 1.29 | 28.03 | 12.62 |

**Table S2.** Occupancies and average number of residues involved in eight different secondary structure motifs.

| | **$TB4^{N\text{-}C}$** | | **$TB4^{N\text{-}mid}$** | | **$TB4^{N\text{-}N}$** | |
|---|---|---|---|---|---|---|
| **Motif** | **(%)** | **n. res.** | **(%)** | **n. res.** | **(%)** | **n. res.** |
| **Random Coil** | 100 | 22.5 | 100 | | 100 | |
| **β-sheet** | 16.8 | 4.4 | 17.6 | 4.4 | 2.5 | |
| **β-bridge** | 62.6 | 2.7 | 52.0 | | 26.1 | |
| **Bend** | 100 | 11.8 | 100 | | 99.9 | |
| **Turn** | 89.3 | 4.8 | 92.7 | | 87.4 | |
| **α-helix** | 18.3 | 4.7 | 26.4 | 5.0 | 26.5 | 4.8 |
| **π-helix** | 0.07 | 5.0 | 0.9 | 5.0 | 0 | 0 |
| **$3_{10}$-helix** | 31.5 | 3.63 | 38.7 | 3.5 | 94.1 | 4.8 |

**Table S3.** Multistep equilibration scheme with decreasing force constants employed in the molecular dynamics study. FC: Force constants. The subscript bb refers to backbone force constants.

| **Equilibration Step** | **$FC_{bb}$ (kJ/mol nm$^2$)** | **NPT/NVT** | **Time (ns)** |
|---|---|---|---|
| Step 1 | 4000.0 | NVT | 0.6 |
| Step 2 | 2000.0 | NVT | 0.6 |
| Step 3 | 1000.0 | NPT | 0.6 |
| Step 4 | 500.0 | NPT | 0.6 |
| Step 5 | 200.0 | NPT | 0.6 |
| Step 6 | 50.0 | NPT | 0.5 |
| Step 7 | 0.0 | NPT | 0.5 |

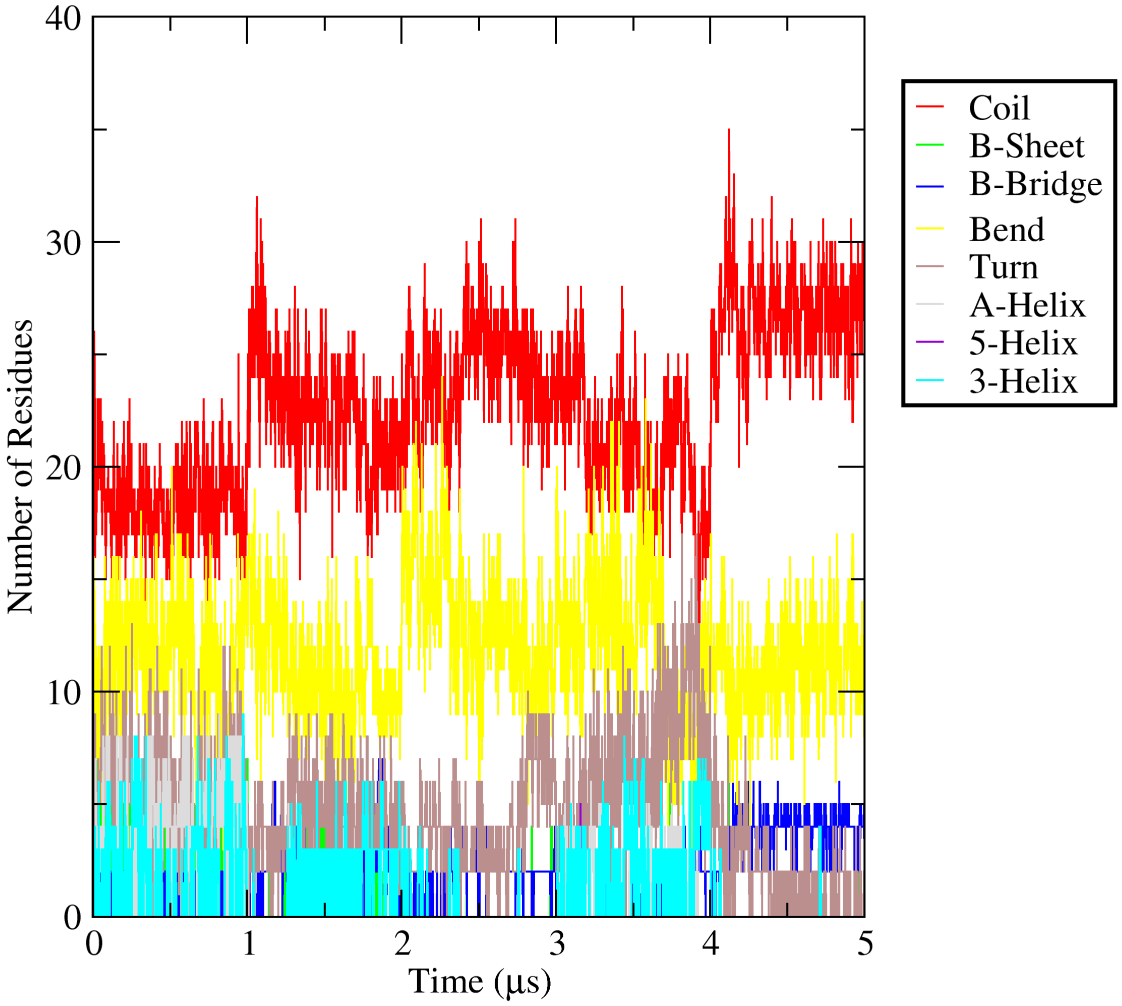


**Figure S16.** Secondary structure analysis according to the DSSP definitions of Tβ4$^{N-C}$.

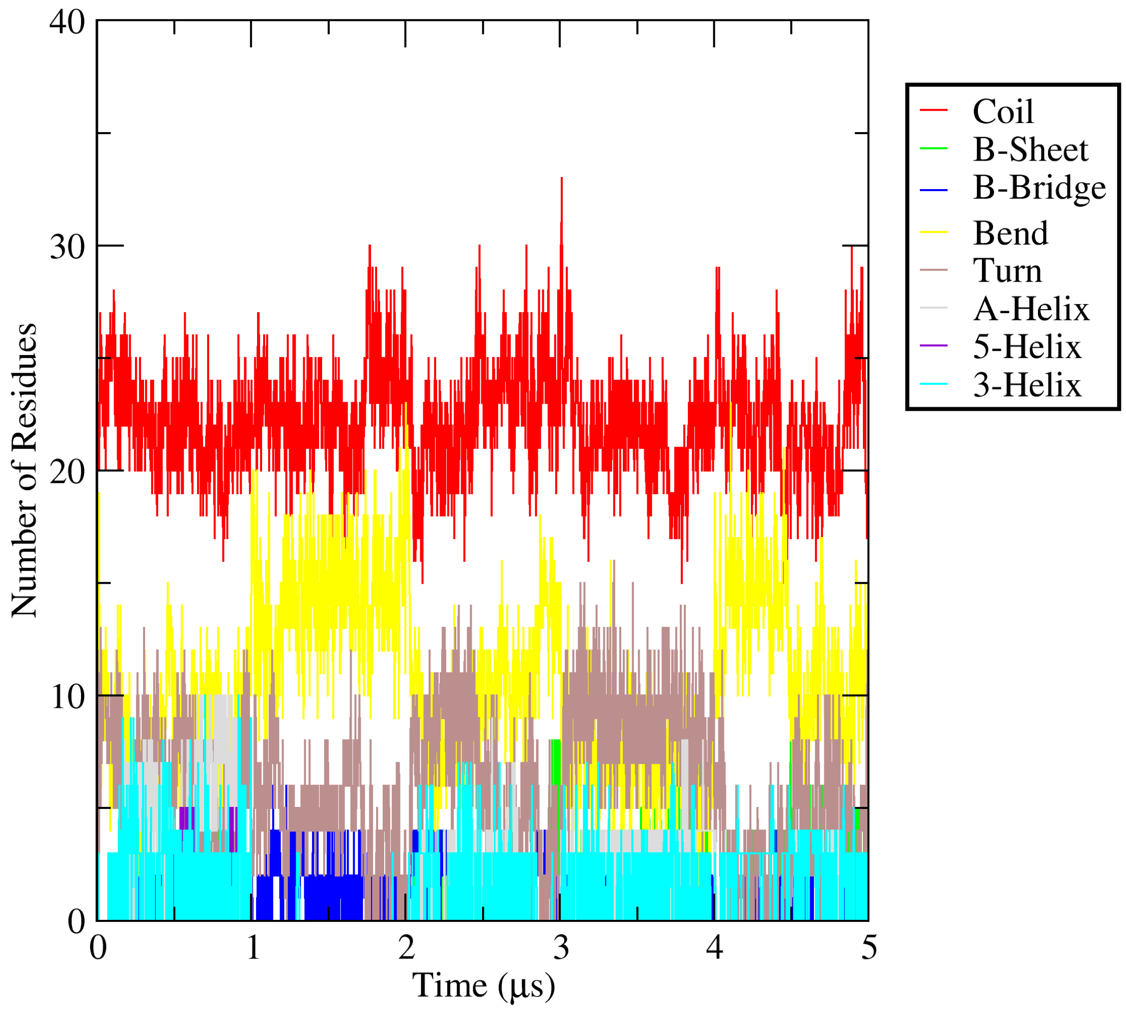


**Figure S17.** Secondary structure analysis according to the DSSP definitions of Tβ4$^{N\text{-}mid}$.

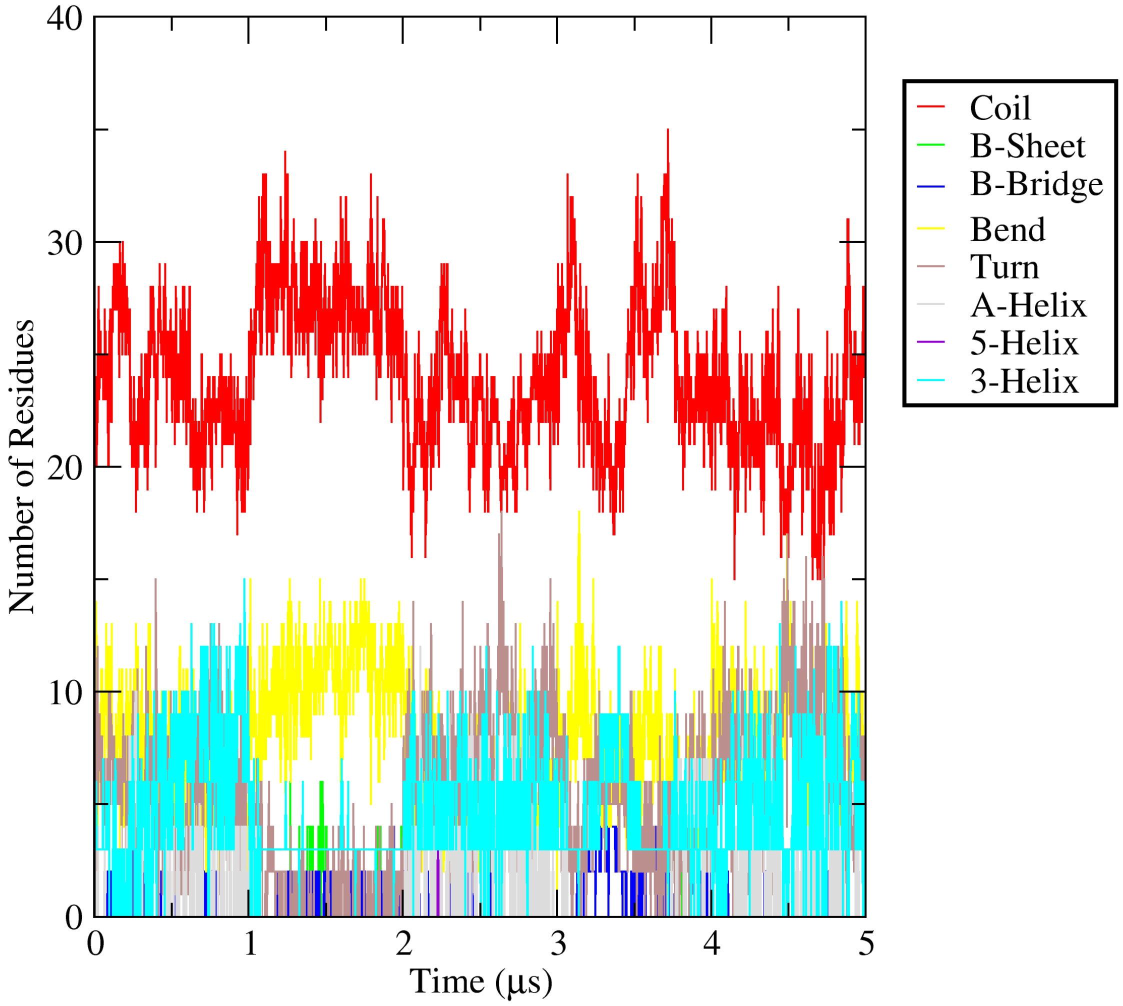


**Figure S18.** Secondary structure analysis according to the DSSP definitions of Tβ4$^{N\text{-}N}$.

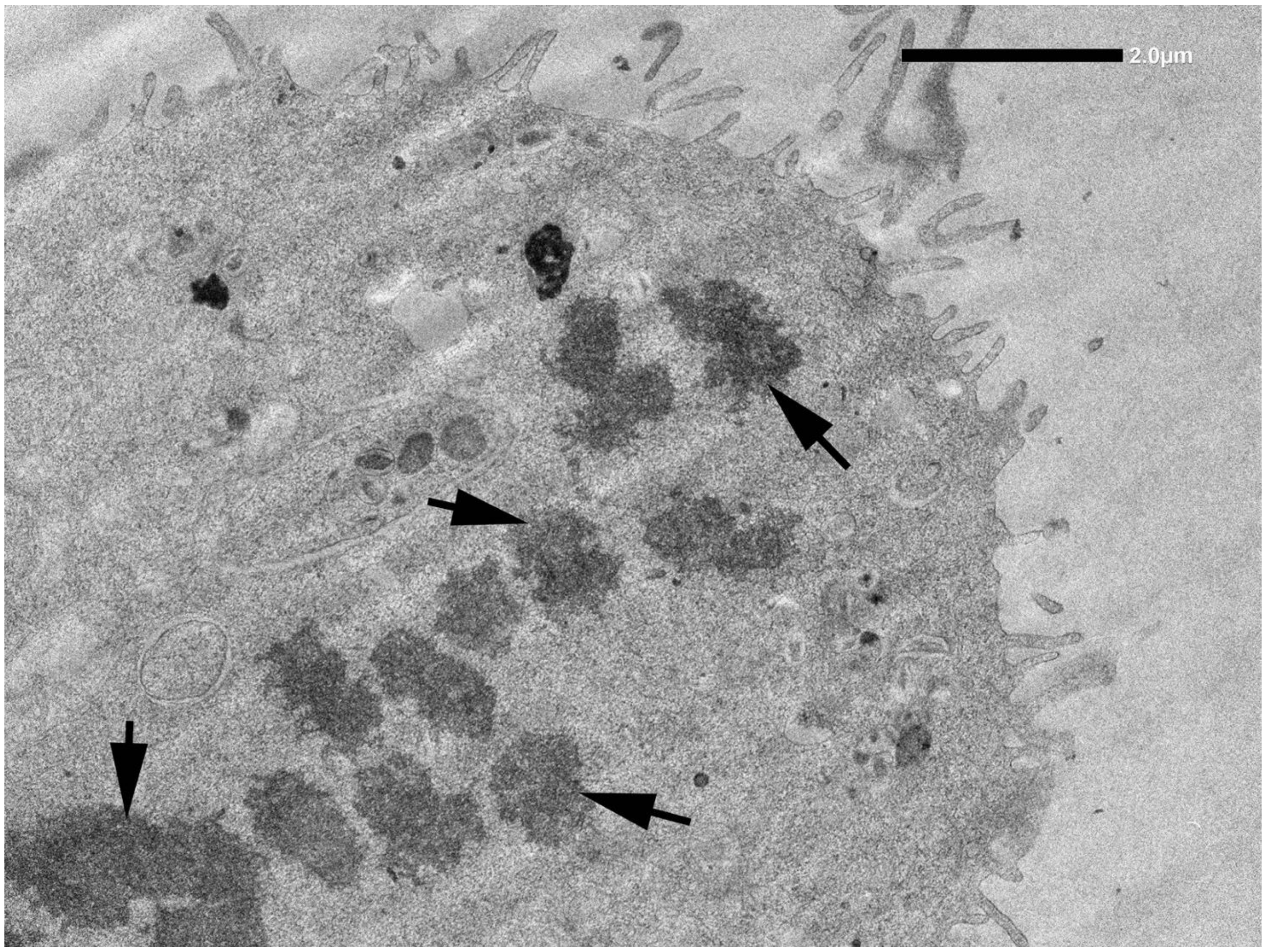


**Figure S19.** Electron micrographs of a mitotic J774 cell. Arrows indicate the condensed packed chromatin of the chromosomes.

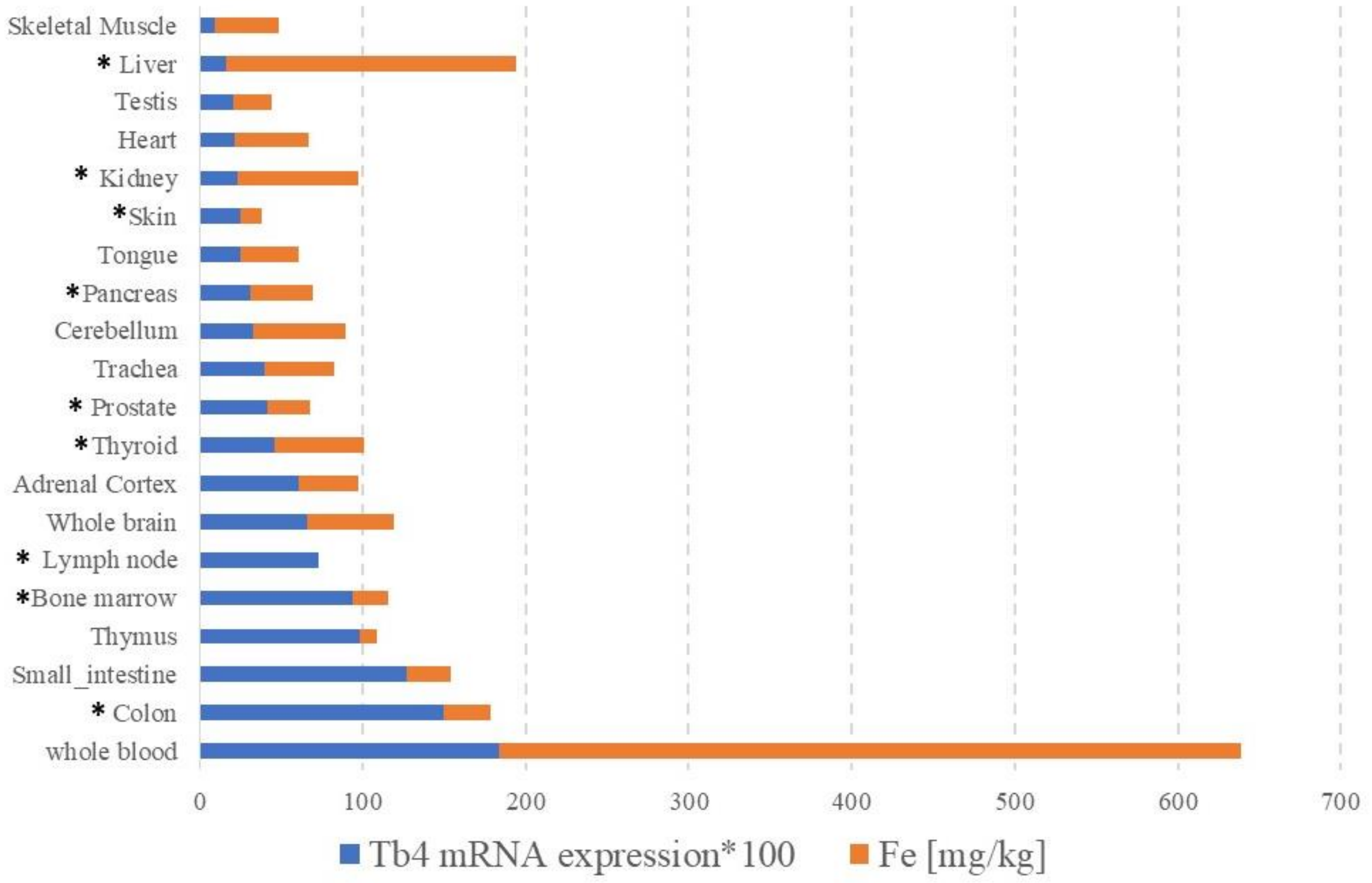


**Scheme S1.** Stacked bar chart listing twenty tissues with the highest Tβ4 mRNA expression (http://biogps.org/) and their iron content[71]. *Tissues with the highest incidence of cancer.

**Table S4.** List of some independent research data and reviews describing Tβ4 and ferroptosis involvement in human pathologies.

| Pathology | Tβ4 involvement | Ferroptosis involvement |
|---|---|---|
| **Cancer** | | |
| Pancreatic | In pancreatic cancer cell lines and in intraductal pancreatic mucinous neoplasms with high grade dysplasia, overexpression of Tβ4 mRNA was found with increased secretion of proinflammatory cytokines[1] | Ferroptosis controls the growth of pancreatic cancer, and the regulatory mechanisms associated with ferroptosis in pancreatic cancer are summarized in a recent review[2]. |
| Hepatocellular carcinoma (HCC) | Immunoreactivity for Tβ4 was detected in 30% of HCC samples. Immunostaining was homogeneous and diffusely distributed over the entire cytoplasm. In the tumor mass, no zonation pattern was observed[3]. | Ferroptosis showed tremendous promise as a therapy, especially in HCC. The role of ferroptosis in HCC, and in the diagnosis and treatment of HCC are summarized in a recent review[4]. |
| Gastric | Increased expression of Tβ4 was observed in 43.3% of gastrointestinal stromal tumors, and significantly associated with tumor size and increased mitosis[5]. | The role of ferroptosis in gastric cancers, and the proteins involved in ferroptosis regulation, were recently reviewed[6]. |
| Colorectal | Tβ4 is overexpressed in a side population of cancer stem cells and CD133-positive colorectal cancer stem cells[7] | Ferroptosis inducer RSL3 initiated cell death and ROS accumulation in HCT116, LoVo, and HT29 CRC cells over a 24 h time course. This effect was reversed by overexpression of GPX4[8]. |
| Breast | The cellular distribution of Tβ4 in breast cancer is heterogenous; multiple cell types within the tumor microenvironment produce Tβ4 and expression varies between tumors[9]. In addition, hypoxia-induced Tß4 is strongly associated with expression of hypoxia inducible factors (HIF-1 and HIF-2) and is also clinicopathologically involved in the lymph node metastatic potential of breast cancer[10]. | Siramesine and Lapatinib treatment induce ferroptosis in breast cancer cells. The ferroptotic process could be used as a new therapeutic strategy to overcome apoptotic resistance in breast cancer[11]. |
| Lung | Tissue microarray analysis showed that Tβ4 was highly expressed in lung cancer[1], and Tβ4 gene silencing in A549 and H1299 cells inhibited cell proliferation, migration, and invasion in vitro, and decreased tumor growth in vivo[12]. | Suppression of NFS1 cooperates with inhibition of cysteine transport to trigger ferroptosis in vitro and slow tumor growth. Therefore, lung adenocarcinomas upregulate pathways that confer resistance to high oxygen tension and protect cells from undergoing ferroptosis in response to oxidative damage[13]. |
| Ovarian | Tβ4 is overexpressed in primary ovarian cancers when compared with normal controls. Tβ4 expression was also co-localized with CD133 expression in primary ovarian carcinomas, metastatic ovarian cancers from stomach cancers, and primary stomach cancers[7]. | Ferroptosis as a new promising anti-tumor strategy in Epithelial ovarian cancer (OVCA), together with various genetic determinants of ferroptosis and their underlying mechanisms in OVCA are described in a recent review[14]. |
| Melanoma | Suppression of Tβ4 expression leads to decreased metastatic potential in murine B16 melanoma cells[15]. Moreover, Tβ4 is crucial for melanoma adhesion and invasion[16]. | Ferroptosis has been described as an efficient strategy in melanoma therapy[17]. |
| Head and neck | Higher TMSB4X expression is found in head and neck squamous cell carcinoma both at the RNA and protein levels. Overexpression of TMSB4X was significantly associated with poor prognosis of overall survival and recurrence-free survival. Silencing of TMSB4X expression in a HNSCC cell line reduced proliferation and invasion ability in vitro, as well as inhibited cervical lymph node metastasis in vivo[18]. | Induction of ferroptosis in head and neck cancer cells overcomes cisplatin resistance[19]. |
| **Neurodegenerative disorders** | | |
| Alzheimer’s Disease | In human Huntington’s and Alzheimer’s disease brains, Tβ4 was found markedly elevated in the cell bodies and processes of | Inhibitors of ferroptosis, such as ferrostatins and liproxstatins, protect from ischemic injury |

| | | |
|---|---|---|
| | reactive microglia distributed to regions of neurodegeneration[20]. | in mouse models in the liver, kidney, brain, and heart[21-26]. These inhibitors are also protective in models of degenerative brain disorders, including Parkinson's, Huntington's, and Alzheimer's diseases, as well as in other forms of neurodegeneration and traumatic and hemorrhagic brain injury[24, 27-33]. |
| Huntington's Disease | The detailed analysis of Huntington's brains revealed that Tβ4 immunoreactivity in reactive microglia was particularly increased during the early stages of pathology[20]. | |
| **Other pathologies** | | |
| Stroke | After injury, Tβ4 is released by platelets, macrophages, and many other cell types to protect cells and tissues from further damage and reduce apoptosis, inflammation, and microbial growth[34]. Tβ4 promotes remodeling of the CNS/PNS post-neural injury and thereby improves neurological recovery[35-38] | The role of ferroptosis in stroke has been recently reviewed[39-41]. |
| Traumatic brain injury (TBI) | Tβ4 may have a role in the repair and remodeling of injured tissues following a hypoxic insult as indicated by a study of focal brain ischemia following occlusion of the middle cerebral artery in rats[42]. Tβ4 may also have a role in neuronal development and remodeling, and<br>transcription of the gene that encodes Tβ4 is upregulated during hypoxia[43] | The role of ferroptosis in TBI is described in recent reviews[44-46]. |
| Myocardial infarction | Administered immediately following a myocardial infarction in mice, Tβ4 protects heart tissue from cell death and enables heart myocytes to survive after hypoxia[47] | GPX4 downregulation during myocardial infarction contributes to ferroptosis in cardiomyocytes[48]. Moreover, ferroptosis is involved in diabetes myocardial ischemia/reperfusion injury through endoplasmic reticulum stress[49] |
| Ischemia/reperfusion (I/R) | Intervention with Tβ4 after ischemia can reduce the neurological deficits in rats[50]. Tβ4 reduced damage after ischemic heart injury, protected post hypoxic cardiac tissue, decreased infarct size, reduced scar volume, decreased inflammation, promoted angiogenesis, and improved ventricular function, and survival[47, 51, 52]. | Inhibitors of ferroptosis, e.g., Galangin[53] and Carvacrol[54]) mitigate ferroptosis and have a neuroprotective effect in I/R. |
| Liver injury and fibrosis | Patients with liver diseases have serum Tβ4 levels negatively correlated with liver function[55]. Exogenous Tβ4 administration ameliorated ischemia reperfusion-induced hepatic injury in mice[56], and prevented in vitro acute liver injury and subsequent fibrosis through alleviating oxidative stress and inflammation[57, 58]. | Ferroptosis plays a crucial role in chronic intermittent hypoxia -induced liver injury[59]. The connection between ferroptosis and non-cancer liver diseases are intricate and compelling[60] |


1. Rebours, V.; Le Faouder, J.; Laouirem, S.; Mebarki, M.; Albuquerque, M.; Camadro, J.-M.; Léger, T.; Ruszniewski, P.; Lévy, P.; Paradis, V., In situ proteomic analysis by MALDI imaging identifies ubiquitin and thymosin-β4 as markers of malignant intraductal pancreatic mucinous neoplasms. *Pancreatology* **2014,** 14, (2), 117-124.
2. Chen, G.; Guo, G.; Zhou, X.; Chen, H., Potential mechanism of ferroptosis in pancreatic cancer. *Oncology letters* **2020,** 19, (1), 579-587.
3. Theunissen, W.; Fanni, D.; Nemolato, S.; Di Felice, E.; Cabras, T.; Gerosa, C.; Van Eyken, P.; Messana, I.; Castagnola, M.; Faa, G., Thymosin beta 4 and thymosin beta 10 expression in hepatocellular carcinoma. *European journal of histochemistry: EJH* **2014,** 58, (1), 2242.
4. Nie, J.; Lin, B.; Zhou, M.; Wu, L.; Zheng, T., Role of ferroptosis in hepatocellular carcinoma. *Journal of cancer research and clinical oncology* **2018,** 144, (12), 2329-2337.
5. Can, B.; Karagoz, F.; Yildiz, L.; Yildirim, A.; Kefeli, M.; Gonullu, G.; Kandemir, B., Thymosin β4 is a novel potential prognostic marker in gastrointestinal stromal tumors. *Apmis* **2012,** 120, (9), 689-698.
6. Song, Y.; Yang, H.; Lin, R.; Jiang, K.; Wang, B. M., The role of ferroptosis in digestive system cancer. *Oncology letters* **2019,** 18, (3), 2159-2164.
7. Ji, Y.-I.; Lee, B.-Y.; Kang, Y.-J.; Jo, J.-O.; Lee, S. H.; Kim, H. Y.; Kim, Y.-O.; Lee, C.; Koh, S. B.; Kim, A., Expression patterns of thymosin β4 and cancer stem cell marker cd133 in ovarian cancers. *Pathology & Oncology Research* **2013,** 19, (2), 237-245.

8. Sui, X.; Zhang, R.; Liu, S.; Duan, T.; Zhai, L.; Zhang, M.; Han, X.; Xiang, Y.; Huang, X.; Lin, H., RSL3 drives ferroptosis through GPX4 inactivation and ROS production in colorectal cancer. *Frontiers in pharmacology* **2018,** 9, 1371.
9. Larsson, L.-I.; Holck, S., Occurrence of thymosin β4 in human breast cancer cells and in other cell types of the tumor microenvironment. *Human pathology* **2007,** 38, (1), 114-119.
10. Yoon, S. Y.; Lee, H. R.; Park, Y.; Kim, J. H.; Kim, S. Y.; Yoon, S. R.; Lee, W. J.; Cho, B. J.; Min, H.; Bang, J.-W., Thymosin β4 expression correlates with lymph node metastasis through hypoxia inducible factor-α induction in breast cancer. *Oncology reports* **2011,** 25, (1), 23-31.
11. Ma, S.; Henson, E.; Chen, Y.; Gibson, S., Ferroptosis is induced following siramesine and lapatinib treatment of breast cancer cells. *Cell death & disease* **2016,** 7, (7), e2307-e2307.
12. Zhang, Y.; Feurino, L. W.; Zhai, Q.; Wang, H.; Fisher, W. E.; Chen, C.; Yao, Q.; Li, M., Thymosin Beta 4 is overexpressed in human pancreatic cancer cells and stimulates proinflammatory cytokine secretion and JNK activation. *Cancer biology & therapy* **2008,** 7, (3), 419-423.
13. Alvarez, S. W.; Sviderskiy, V. O.; Terzi, E. M.; Papagiannakopoulos, T.; Moreira, A. L.; Adams, S.; Sabatini, D. M.; Birsoy, K.; Possemato, R., NFS1 undergoes positive selection in lung tumours and protects cells from ferroptosis. *nature* **2017,** 551, (7682), 639-643.
14. Lin, C.-C.; Chi, J.-T., Ferroptosis of epithelial ovarian cancer: genetic determinants and therapeutic potential. *Oncotarget* **2020,** 11, (39), 3562.
15. Kim, A.; Son, M.; Kim, K. I.; Yang, Y.; Song, E. Y.; Lee, H. G.; Lim, J.-S., Elevation of intracellular cyclic AMP inhibits NF-κB-mediated thymosin β4 expression in melanoma cells. *Experimental cell research* **2009,** 315, (19), 3325-3335.
16. Makowiecka, A.; Malek, N.; Mazurkiewicz, E.; Mrówczyńska, E.; Nowak, D.; Mazur, A. J., Thymosin β4 regulates focal adhesion formation in human melanoma cells and affects their migration and invasion. *Frontiers in cell and developmental biology* **2019,** 7, 304.
17. Gagliardi, M.; Saverio, V.; Monzani, R.; Ferrari, E.; Piacentini, M.; Corazzari, M., Ferroptosis: a new unexpected chance to treat metastatic melanoma? *Cell Cycle* **2020,** 19, (19), 2411-2425.
18. Chi, L.-H.; Chang, W.-M.; Chang, Y.-C.; Chan, Y.-C.; Tai, C.-C.; Leung, K.-W.; Chen, C.-L.; Wu, A. T.; Lai, T.-C.; Li, Y.-C. J., Global proteomics-based identification and validation of thymosin Beta-4 X-linked as a prognostic marker for head and neck squamous cell carcinoma. *Scientific reports* **2017,** 7, (1), 1-13.
19. Roh, J.-L.; Kim, E. H.; Jang, H. J.; Park, J. Y.; Shin, D., Induction of ferroptotic cell death for overcoming cisplatin resistance of head and neck cancer. *Cancer letters* **2016,** 381, (1), 96-103.
20. Sapp, E.; Kegel, K.; Aronin, N.; Hashikawa, T.; Uchiyama, Y.; Tohyama, K.; Bhide, P.; Vonsattel, J.; DiFiglia, M., Early and progressive accumulation of reactive microglia in the Huntington disease brain. *Journal of Neuropathology & Experimental Neurology* **2001,** 60, (2), 161-172.
21. Angeli, J. P. F.; Schneider, M.; Proneth, B.; Tyurina, Y. Y.; Tyurin, V. A.; Hammond, V. J.; Herbach, N.; Aichler, M.; Walch, A.; Eggenhofer, E., Inactivation of the ferroptosis regulator Gpx4 triggers acute renal failure in mice. *Nature cell biology* **2014,** 16, (12), 1180-1191.
22. Gao, M.; Monian, P.; Quadri, N.; Ramasamy, R.; Jiang, X., Glutaminolysis and transferrin regulate ferroptosis. *Molecular cell* **2015,** 59, (2), 298-308.
23. Linkermann, A.; Skouta, R.; Himmerkus, N.; Mulay, S. R.; Dewitz, C.; De Zen, F.; Prokai, A.; Zuchtriegel, G.; Krombach, F.; Welz, P.-S., Synchronized renal tubular cell death involves ferroptosis. *Proceedings of the National Academy of Sciences* **2014,** 111, (47), 16836-16841.
24. Skouta, R.; Dixon, S. J.; Wang, J.; Dunn, D. E.; Orman, M.; Shimada, K.; Rosenberg, P. A.; Lo, D. C.; Weinberg, J. M.; Linkermann, A., Ferrostatins inhibit oxidative lipid damage and cell death in diverse disease models. *Journal of the American Chemical Society* **2014,** 136, (12), 4551-4556.
25. Tuo, Q.; Lei, P.; Jackman, K.; Li, X.; Xiong, H.; Liuyang, Z.; Roisman, L.; Zhang, S.; Ayton, S.; Wang, Q., Tau-mediated iron export prevents ferroptotic damage after ischemic stroke. *Molecular psychiatry* **2017,** 22, (11), 1520-1530.
26. Dixon, S. J.; Lemberg, K. M.; Lamprecht, M. R.; Skouta, R.; Zaitsev, E. M.; Gleason, C. E.; Patel, D. N.; Bauer, A. J.; Cantley, A. M.; Yang, W. S., Ferroptosis: an iron-dependent form of nonapoptotic cell death. *Cell* **2012,** 149, (5), 1060-1072.
27. Chen, L.; Hambright, W. S.; Na, R.; Ran, Q., Ablation of the ferroptosis inhibitor glutathione peroxidase 4 in neurons results in rapid motor neuron degeneration and paralysis. *Journal of Biological Chemistry* **2015,** 290, (47), 28097-28106.
28. Do Van, B.; Gouel, F.; Jonneaux, A.; Timmerman, K.; Gelé, P.; Pétrault, M.; Bastide, M.; Laloux, C.; Moreau, C.; Bordet, R., Ferroptosis, a newly characterized form of cell death in Parkinson's disease that is regulated by PKC. *Neurobiology of disease* **2016,** 94, 169-178.
29. Gascón, S.; Murenu, E.; Masserdotti, G.; Ortega, F.; Russo, G. L.; Petrik, D.; Deshpande, A.; Heinrich, C.; Karow, M.; Robertson, S. P., Identification and successful negotiation of a metabolic checkpoint in direct neuronal reprogramming. *Cell stem cell* **2016,** 18, (3), 396-409.
30. Guiney, S. J.; Adlard, P. A.; Bush, A. I.; Finkelstein, D. I.; Ayton, S., Ferroptosis and cell death mechanisms in Parkinson's disease. *Neurochemistry international* **2017,** 104, 34-48.
31. Hambright, W. S.; Fonseca, R. S.; Chen, L.; Na, R.; Ran, Q., Ablation of ferroptosis regulator glutathione peroxidase 4 in forebrain neurons promotes cognitive impairment and neurodegeneration. *Redox biology* **2017,** 12, 8-17.
32. Li, Q.; Han, X.; Lan, X.; Gao, Y.; Wan, J.; Durham, F.; Cheng, T.; Yang, J.; Wang, Z.; Jiang, C., Inhibition of neuronal ferroptosis protects hemorrhagic brain. *JCI insight* **2017,** 2, (7).
33. Zille, M.; Karuppagounder, S. S.; Chen, Y.; Gough, P. J.; Bertin, J.; Finger, J.; Milner, T. A.; Jonas, E. A.; Ratan, R. R., Neuronal death after hemorrhagic stroke in vitro and in vivo shares features of ferroptosis and necroptosis. *Stroke* **2017,** 48, (4), 1033-1043.
34. Goldstein, A. L.; Hannappel, E.; Sosne, G.; Kleinman, H. K., Thymosin β4: a multi-functional regenerative peptide. Basic properties and clinical applications. *Expert opinion on biological therapy* **2012,** 12, (1), 37-51.
35. Morris, D. C.; Chopp, M.; Zhang, L.; Lu, M.; Zhang, Z. G., Thymosin β4 improves functional neurological outcome in a rat model of embolic stroke. *Neuroscience* **2010,** 169, (2), 674-682.
36. Xiong, Y.; Mahmood, A.; Meng, Y.; Zhang, Y.; Zhang, Z. G.; Morris, D. C.; Chopp, M., Treatment of traumatic brain injury with thymosin β4 in rats. *Journal of neurosurgery* **2011,** 114, (1), 102-115.

37. Zhang, J.; Zhang, Z. G.; Morris, D.; Li, Y.; Roberts, C.; Elias, S. B.; Chopp, M., Neurological functional recovery after thymosin beta4 treatment in mice with experimental auto encephalomyelitis. *Neuroscience* **2009,** 164, (4), 1887-1893.
38. Wang, L.; Chopp, M.; Szalad, A.; Liu, Z.; Lu, M.; Zhang, L.; Zhang, J.; Zhang, R. L.; Morris, D.; Zhang, Z. G., Thymosin β4 promotes the recovery of peripheral neuropathy in type II diabetic mice. *Neurobiology of disease* **2012,** 48, (3), 546-555.
39. DeGregorio-Rocasolano, N.; Martí-Sistac, O.; Gasull, T., Deciphering the iron side of stroke: neurodegeneration at the crossroads between iron dyshomeostasis, excitotoxicity, and ferroptosis. *Frontiers in neuroscience* **2019,** 13, 85.
40. Ren, J.-X.; Sun, X.; Yan, X.-L.; Guo, Z.-N.; Yang, Y., Ferroptosis in neurological diseases. *Frontiers in Cellular Neuroscience* **2020,** 14.
41. Weiland, A.; Wang, Y.; Wu, W.; Lan, X.; Han, X.; Li, Q.; Wang, J., Ferroptosis and its role in diverse brain diseases. *Molecular Neurobiology* **2019,** 56, (7), 4880-4893.
42. Vartiainen, N.; Pyykönen, I.; Hökfelt, T.; Koistinaho, J., Induction of thymosin beta (4) mRNA following focal brain ischemia. *Neuroreport* **1996,** 7, (10), 1613-1616.
43. Wang, J.-H.; Shan, Y.-J.; Cong, Y.-W.; Wu, L.-J.; Yuan, X.-L.; Zhao, Z.-H.; Wang, S.-Q.; Chen, J.-P., Identification of differentially expressed genes of acute hypoxia-treated HepG2 cells and hypoxia-acclimatized HepG2 cells. *Sheng li xue bao:[Acta physiologica Sinica]* **2003,** 55, (3), 324-330.
44. Kenny, E. M.; Fidan, E.; Yang, Q.; Anthonymuthu, T. S.; New, L. A.; Meyer, E. A.; Wang, H.; Kochanek, P. M.; Dixon, C. E.; Kagan, V. E., Ferroptosis contributes to neuronal death and functional outcome after traumatic brain injury. *Critical care medicine* **2019,** 47, (3), 410.
45. Magtanong, L.; Dixon, S. J., Ferroptosis and brain injury. *Developmental neuroscience* **2018,** 40, (5-6), 382-395.
46. Tang, S.; Gao, P.; Chen, H.; Zhou, X.; Ou, Y.; He, Y., The Role of Iron, Its Metabolism and Ferroptosis in Traumatic Brain Injury. *Frontiers in Cellular Neuroscience* **2020,** 14, 317.
47. Bock-Marquette, I.; Saxena, A.; White, M. D.; DiMaio, J. M.; Srivastava, D., Thymosin β4 activates integrin-linked kinase and promotes cardiac cell migration, survival and cardiac repair. *nature* **2004,** 432, (7016), 466-472.
48. Park, T.-J.; Park, J. H.; Lee, G. S.; Lee, J.-Y.; Shin, J. H.; Kim, M. W.; Kim, Y. S.; Kim, J.-Y.; Oh, K.-J.; Han, B.-S., Quantitative proteomic analyses reveal that GPX4 downregulation during myocardial infarction contributes to ferroptosis in cardiomyocytes. *Cell death & disease* **2019,** 10, (11), 1-15.
49. Li, W.; Li, W.; Leng, Y.; Xiong, Y.; Xia, Z., Ferroptosis is involved in diabetes myocardial ischemia/reperfusion injury through endoplasmic reticulum stress. *DNA and Cell Biology* **2020,** 39, (2), 210-225.
50. Zhang, Z.; Liu, S.; Huang, S., Effects of thymosin β4 on neuronal apoptosis in a rat model of cerebral ischemia-reperfusion injury. *Molecular medicine reports* **2019,** 20, (5), 4186-4192.
51. Crockford, D., Development of thymosin β4 for treatment of patients with ischemic heart disease. *Annals of the New York Academy of Sciences* **2007,** 1112, (1), 385-395.
52. El-Aouni, C.; Olson, T.; Horstkotte, J.; Mayer, S.; Muller, S.; Willhauck, M.; Spitzweg, C.; Gildehaus, F. J.; Munzing, W.; Hannappel, E.; Bock-Marquette, I.; DiMaio, J. M.; Hatzopoulos, A. K.; Boekstegers, P.; Kupatt, C., Thymosin beta4 is an essential paracrine factor of embryonic endothelial progenitor cell-mediated cardioprotection. *Circulation* **2008,** 117, (17), 2232-2240.
53. Guan, X.; Li, Z.; Zhu, S.; Cheng, M.; Ju, Y.; Ren, L.; Yang, G.; Min, D., Galangin attenuated cerebral ischemia reperfusion injury by inhibition of ferroptosis through activating the SLC7A11/GPX4 axis in gerbils. *Life sciences* **2021,** 264, 118660.
54. Guan, X.; Li, X.; Yang, X.; Yan, J.; Shi, P.; Ba, L.; Cao, Y.; Wang, P., The neuroprotective effects of carvacrol on ischemia/reperfusion-induced hippocampal neuronal impairment by ferroptosis mitigation. *Life sciences* **2019,** 235, 116795.
55. Xing, J.; HAN, T.; ZHU, Z.-y., Serum thymosin β4 level in patients with hepatocellular carcinoma. *Journal of clinical hepatology* **2011,** 27, 387-390.
56. Li, X.; Feng, B.; Shen, J.; Gui, L.; Wang, M.; Xu, Y., Thymosinβ4 Alleviates Mouse Liver Ischemia-Reperfusion Injury by Activating AKT-Bad Pathway.: Abstract# P-23. *Liver Transplantation* **2012,** 18.
57. Li, X.; Wang, L.; Chen, C., Effects of exogenous thymosin β4 on carbon tetrachloride-induced liver injury and fibrosis. *Scientific reports* **2017,** 7, (1), 1-13.
58. Shah, R.; Reyes-Gordillo, K.; Cheng, Y.; Varatharajalu, R.; Ibrahim, J.; Lakshman, M. R., Thymosin β4 prevents oxidative stress, inflammation, and fibrosis in ethanol-and LPS-induced liver injury in mice. *Oxidative medicine and cellular longevity* **2018,** 2018.
59. Chen, L.-D.; Wu, R.-H.; Huang, Y.-Z.; Chen, M.-X.; Zeng, A.-M.; Zhuo, G.-f.; Xu, F.-S.; Liao, R.; Lin, Q.-C., The role of ferroptosis in chronic intermittent hypoxia-induced liver injury in rats. *Sleep and Breathing* **2020**, 1-7.
60. Mao, L.; Zhao, T.; Song, Y.; Lin, L.; Fan, X.; Cui, B.; Feng, H.; Wang, X.; Yu, Q.; Zhang, J., The emerging role of ferroptosis in non-cancer liver diseases: hype or increasing hope? *Cell death & disease* **2020,** 11, (7), 1-12.
